# Integrated machine learning and positive matrix factorization for the source-specific contamination and predictive risk assessment of potentially toxic elements in multi-land-use soils around an active coal mine

**DOI:** 10.1039/d5ra09789d

**Published:** 2026-02-19

**Authors:** Zahid Bashir, Deep Raj, Rangabhashiyam Selvasembian

**Affiliations:** a Department of Environmental Science and Engineering, School of Engineering and Sciences, SRM University-AP Amaravati Andhra Pradesh 522240 India Zahidbashir5175@gmail.com deepraj2587@gmail.com rambhashiyam@gmail.com; b Centre for Interdisciplinary Research, SRM University-AP Amaravati Andhra Pradesh 522240 India

## Abstract

Investigating the distribution, sources, and risks of potentially toxic elements (PTEs) in mining-impacted soils is critical for effective environmental monitoring and human health protection. However, traditional assessments often fail to integrate spatial, source-oriented, and predictive approaches limiting a comprehensive understanding. In this study, 120 soil samples were collected from five land-use types surrounding an active opencast coal mine in the Godavari Valley coalfields, India. Pollution indices revealed severe multi-metal contamination, with Co and Cd emerging as the most consistently enriched elements across land uses, while Zn showed pronounced but spatially restricted enrichment, particularly in coal mine soils. An integrated framework combining positive matrix factorization (PMF), machine learning, and geospatial analysis was developed to identify source-specific contamination patterns. A robust four-factor PMF solution identified mixed industrial-mining activities as the dominant source (∼49%) of contamination. A random forest (RF) model integrating soil properties, spatial variables, and PMF-derived source contributions demonstrated strong to moderate predictive performance (average *R*^2^ = 0.82) with an average root mean square error (RMSE) of 19.6 mg kg^−1^. Geostatistical mapping highlighted coal mines and adjacent agricultural areas as persistent contamination hotspots. Ecological risk assessment indicated Cd and Hg as the principal contributors to high ecological risks, particularly in agricultural and roadside soils. Probabilistic health risk assessment revealed unacceptable risks for the local population, with children being the most vulnerable. Cr was identified as the primary driver of carcinogenic risk, contributing ∼81% in children, while Co-dominated non-carcinogenic risks resulting in hazard indices for children approaching unacceptable thresholds across all land-uses. Our findings provide a precise and scientific framework for source-specific risk assessment to target soil remediation and environmental management in mining-impacted landscapes worldwide.

## Abstract 2

Using geospatial analysis, receptor modelling, and machine learning, this study evaluates potentially toxic element contamination across land uses near an active coal mine, linking distribution, sources, and risk assessment.

## Graphical abstract

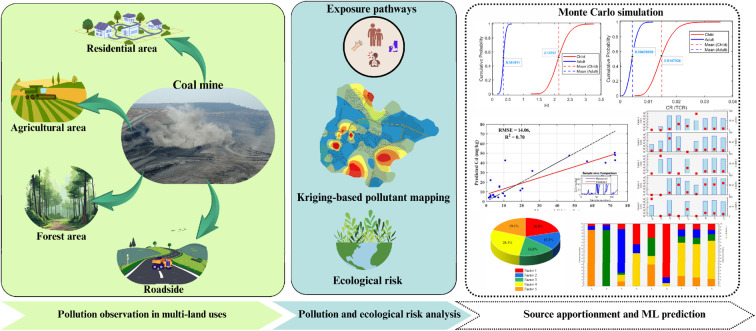

## 1. Introduction

The widespread contamination of soils by potentially toxic elements (PTEs) represents a critical environmental issue worldwide. This contamination is predominantly driven by anthropogenic activities such as mining, metallurgical operations, and chemical production.^1,2^ PTEs persist in the environment, causing long-term soil contamination and ecological impacts. The persistence of PTEs facilitates their uptake by crops and subsequent bioaccumulation, thereby introducing these toxicants into the food chain.^3^ Prolonged human exposure to PTEs has been epidemiologically linked to a spectrum of chronic health complications, including renal, pulmonary, and cardiovascular disorders.^2,4^

Although fundamental to global energy production, coal contains elevated levels of trace and major PTEs.^5^ The processes of extraction, processing, and transportation release substantial amounts of particulate matter, which acts as vectors for the dispersal of PTEs into surrounding ecosystems.^6,7^ Consequently, soils in coal mining areas exhibit elevated PTE concentrations.^8^ This phenomenon is documented globally. For instance, in the Raniganj coalfield in India, soils have been reported to contain high concentrations of arsenic (As) (9.56 mg kg^−1^), chromium (Cr) (265.4 mg kg^−1^), and lead (Pb) (122.7 mg kg^−1^).^9^ A broader global study by Sahoo *et al.*^10^ confirmed extensive contamination, reporting wide concentration ranges for key PTEs such as Pb (0.5–433 mg kg^−1^), Cr (17.5–954 mg kg^−1^), and Ni (4.3–390 mg kg^−1^), in coal mining-affected soils. Similarly, significant PTE enrichment has been recorded in diverse geographical contexts, including a coal-mine brownfield in Beijing, China^11^ and the abandoned Enugu coal mine in Nigeria, where extreme levels of Zn (353.34 mg kg^−1^) and As (58.83 mg kg^−1^) have been detected.^12^

Soil contamination is a complex process influenced by both natural and human-driven factors.^13^ Therefore, the accurate apportionment of contamination sources is critical for devising targeted and effective pollution control methods. Receptor modelling tools, such as positive matrix factorization (PMF), provide a robust framework for distinguishing between geogenic and anthropogenic contributions to soil contamination.^14^ The PMF model decomposes a multivariate dataset of contaminant concentrations into a set of non-negative latent source factors based on their correlation and covariance structure.^15^ A key strength of this approach is its incorporation of measurement uncertainty, which allows it to effectively handle missing or imprecise data while yielding geochemically explainable source profiles.^16^ Owing to these capabilities, PMF has been commonly adopted for source apportionment across diverse ecological compartments, including air, soil, water and sediments.^17^ Beyond the traditional analysis of factor loadings, recent applications have highlighted the value of spatially visualizing the contributions of PMF-derived factors. This advanced approach provides a more interpretable understanding of pollution source distribution.^17–19^

The physicochemical properties of soil and nutrient levels are fundamental in controlling the behaviour and availability of PTEs in the environment. However, conventional methods for directly measuring the PTE concentrations are laborious and time-consuming, creating the need for more efficient assessment tools. In response, machine learning (ML) approaches, particularly the random forest (RF) algorithm, have increasingly been used for predicting PTE concentrations by modelling complex, nonlinear relationships with environmental predictors.^20,21^ However, such models frequently lack direct integration with quantitative pollution source data, limiting their ability to validate and explain the underlying drivers of contamination. Furthermore, to reliably convert measured contamination levels into actionable public health insights, it is essential to move beyond deterministic calculations. Employing probabilistic methods such as Monte Carlo simulation for health risk assessment is critical, as this approach quantifies uncertainty and variability in exposure parameters, providing a more realistic evaluation of both carcinogenic and non-carcinogenic risks to vulnerable populations.^22,23^

Despite the extensive research on PTE contamination, a key methodological gap persists. Most studies treat geostatistics, source apportionment, and predictive modelling as separate tools, reducing their ability to capture the complex and highly variable contamination patterns. This shortcoming is particularly critical in countries such as India, where extensive mining activity and diverse land-use conditions intensify the spatial variability. Therefore, an integrated framework was applied to improve the spatial characterization of pollution sources and the prediction of PTE concentrations in the Jalagam Vengal Rao (JVR) opencast (OC) coal mine located in the South Godavari Valley coalfields. The specific objectives of this study were to: (i) map the spatial distribution of PTEs (mercury (Hg), arsenic (As), zinc (Zn), lead (Pb), cobalt (Co), cadmium (Cd), nickel (Ni), chromium (Cr), and copper (Cu)) across diverse land uses using ordinary kriging interpolation; (ii) apportion contamination sources using PMF and map their spatial dominance through interpolation of PMF-derived factor contributions; (iii) validate an RF model for predicting and forecasting PTE concentrations, and (iv) evaluate ecological risk using established pollution indices and assess human health risks using probabilistic and deterministic approaches. The findings offer actionable insights for targeted soil remediation and risk mitigation in coal mining-affected areas and demonstrate the applicability of geospatial assessment methods to similar environments worldwide.

## 2. Materials and methods

### 2.1 Description of the study area

**Fig. 1:**
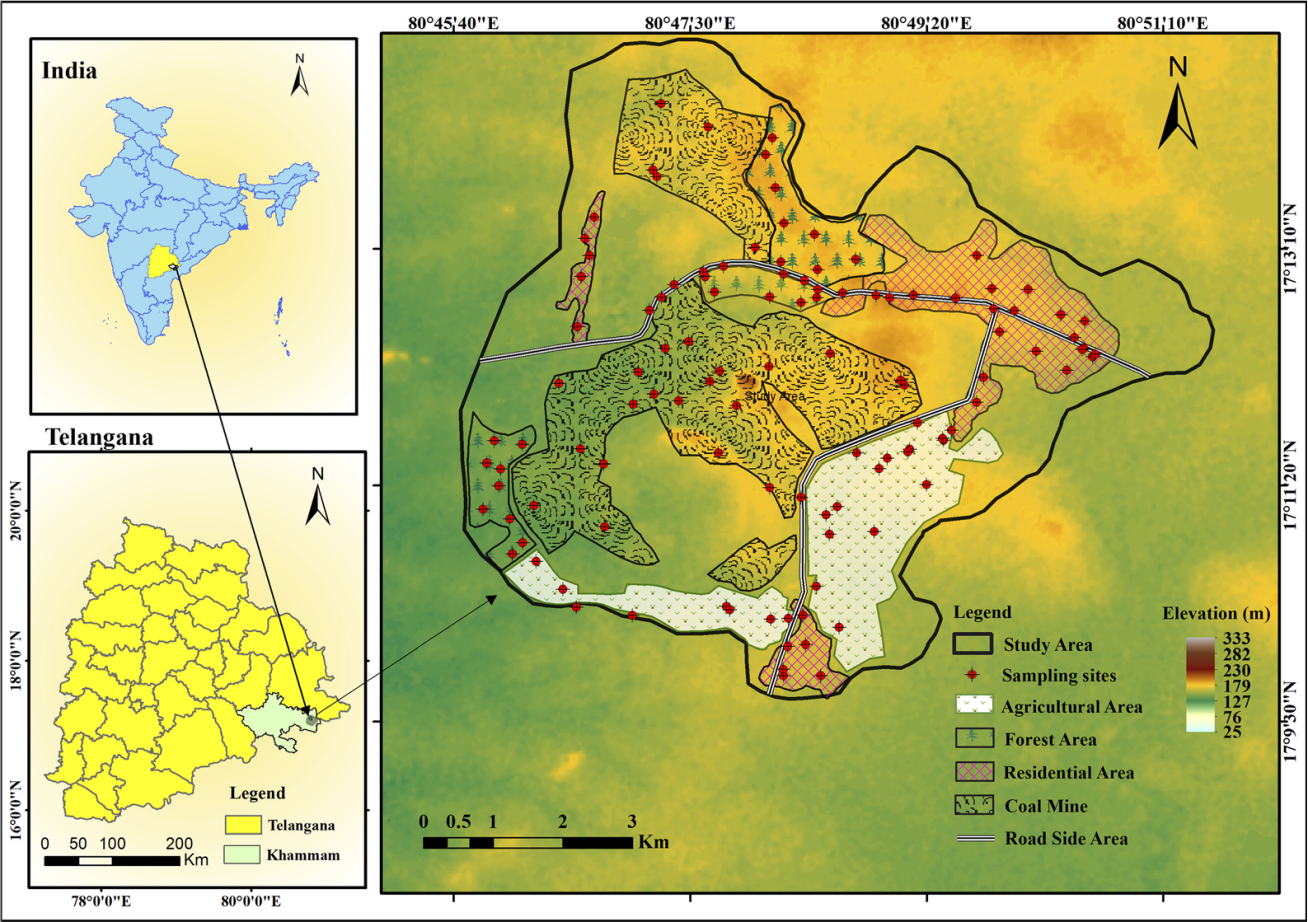
Study area map showing different land use types and the spatial distribution of soil sampling locations.

The Godavari Valley coalfields span nearly 9000 km^2^ and are divided into 12 major coal-bearing zones. Mining activity is extensive across this region, with 15 opencast and 35 underground mines currently in operation. The present investigation focuses on the JVR mining zone in the Khammam district of Telangana (17°12′22.07″N, 80°47′59.24″E) (Fig. 1), which forms an integral segment of the larger Godavari Valley coal belt. The area is characterized by a tropical monsoon climate experiencing warm summers (40–45 °C) and mild winters (18–25 °C). Rainfall in this region averages between 800 and 1100 mm per year. The terrain is undulating and forms part of the south-eastern Deccan Plateau. The geological formations primarily comprise Gondwana rock strata, which are rich in coal deposits. The local geology is dominated by sedimentary strata of the Barakar Formation, consisting mainly of sandstone, shale, and coal seams.^24,25^

The JVR mine produces substantial quantities of non-coking, Grade G, sub-bituminous coal. The coal seams present in this area vary in width but are near the surface, making open-pit mining the primary extraction method. Currently, the opencast project (JVR I & II Expansion) has an annual output of around 4 million tonnes of coal. Plans are underway to increase production substantially, targeting an eventual capacity of 10 million tonnes annually. The operation is projected to recover nearly 245.51 million tonnes of coal over an estimated lifespan of 28 years.

The coal mine is surrounded by different land use types, including coal mine (CM) (JVR OC I & II and Kistaram OC), agricultural (AG), residential (RD), forest (FT), and roadside (RS) areas (Fig. 1). The study area and land use boundaries were delineated using Google Earth Pro, and relevant spatial data were imported into ArcGIS (ArcMap 10.8) for mapping.

### 2.2 Soil sample collection and analysis

In this study, a total of 120 soil samples were collected from a depth of 0–20 cm using a stratified random sampling approach. To ensure proportionate representation of the site conditions, 24 samples were obtained from each of the five land-use categories, CM, AG, RD, FT, and RS. A composite sample was prepared from three randomly drawn subsamples, all collected within a 20 m radius of the central sampling point. A stainless-steel auger was employed for soil extraction, and samples were subsequently sealed in plastic bags for transport to the laboratory. Upon receipt at the laboratory, the samples underwent natural air-drying. The samples were processed by grinding and subsequently sieving through a 100-mesh screen to achieve a uniform particle size.

Soil pH and electrical conductivity (EC) were measured in a 1 : 2.5 soil/deionized water suspension using a multi-parameter probe (HANNA HI98194). The total carbon (C%) and nitrogen (N%) contents in the soil were determined through a CHNS analyzer (UNICUBE®). Cation exchange capacity (CEC) was quantified through ammonium acetate saturation at pH 7, followed by the displacement and colorimetric analysis of NH_4_^+^ ions.^26^ Soil organic matter (OM%) was measured by the loss-on-ignition method at 550 °C and soil texture was determined using the hydrometer method.^27^

For PTE analysis, a 0.5 g aliquot of homogenized soil was digested using the USEPA 3050B and 6020B methods,^28^ involving sequential treatment with concentrated HNO_3_, H_2_O_2_, and HCl to achieve complete matrix dissolution. The concentrations of Hg, As, Zn, Pb, Co, Cd, Ni, Cr, and Cu were then measured by inductively coupled plasma-optical emission spectrometry (ICP-OES) (iCAP™ PRO X Duo ICP-OES).

### 2.3 Quality assurance (QA) and quality control (QC)

QA and QC methods were applied throughout the digestion and analytical processes. Triplicate analyses and method blanks were included with each batch of samples to assess precision and contamination. Analytical accuracy was verified using the certified reference material MESS-4 (marine sediment certified reference material for total and extractable metal content) obtained from the National Research Council of Canada, along with matrix-spiked samples. The recoveries for all analyzed PTEs ranged from 92% to 109%. Instrument calibration of the ICP-OES was carried out using a multi-element standard solution (Inorganic Ventures, FINAR-92), and measurement precision was maintained within a relative standard deviation of ±5%. The method detection limits based on replicate analyses of procedural blanks and accounting for the complete digestion and analytical process for the analysed elements were 0.002 mg kg^−1^ for Cr, Cu, and Ni; 0.0027 mg kg^−1^ for Co, 0.003 mg kg^−1^ for As; 0.005 mg kg^−1^ for Hg; 0.01 mg kg^−1^ for Zn, 0.015 mg kg^−1^ for Cd; and 0.021 mg kg^−1^ for Pb.

### 2.4 Pollution indices

To comprehensively evaluate PTE contamination and its ecological implications, a group of established pollution indices was employed. The contamination factor (CF) was calculated to determine the enrichment level of individual metals relative to background values. The geo-accumulation index (*I*_geo_) further refines this assessment using a logarithmic scale to classify the contamination levels, effectively distinguishing between natural variability and anthropogenic contributions.^29^ The pollution load index (PLI) was then used to integrate the individual contamination factors into an overall measure of site pollution. Furthermore, the potential ecological impact was assessed using the ecological risk factor (ER) for individual metals and the potential ecological risk index (PERI), which aggregates multiple ER values to reflect the cumulative toxicological stress on the ecosystem.^29,30^ The calculation formulas are provided below:1
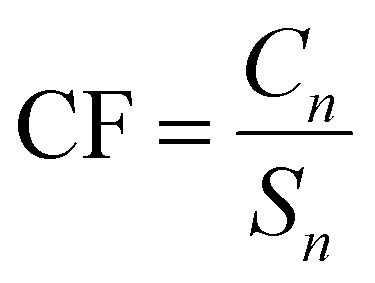
2
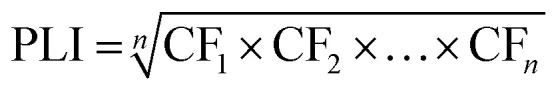
3
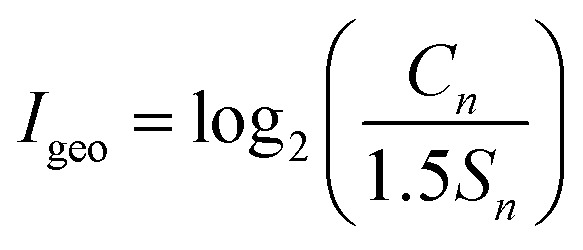

4 ER = *T*_r_^*i*^ × CF

5
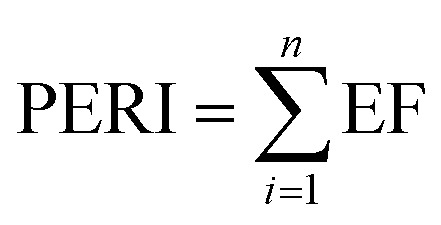


Based on the pollution severity, the classification levels of CF, PLI, *I*_geo_, ER and PERI are provided in Table S1.

### 2.5 Spatial distribution of potentially toxic elements

The spatial distribution of PTE concentrations across the study area was modelled using the ordinary kriging interpolation method. This method leverages the spatial dependence characterized by a semivariogram model to calculate the optimal weights for surrounding unsampled data points. The predicted value at an unsampled location (*x*_0_) is derived as a weighted sum of measured values [eqn (6)], making it a powerful complement to the stratified random sampling design for generating continuous prediction maps. The experimental semivariograms for all elements were best fitted using spherical models, and the corresponding nugget, sill, and range parameters applied in the kriging analysis are presented in the SI (Table S9).6
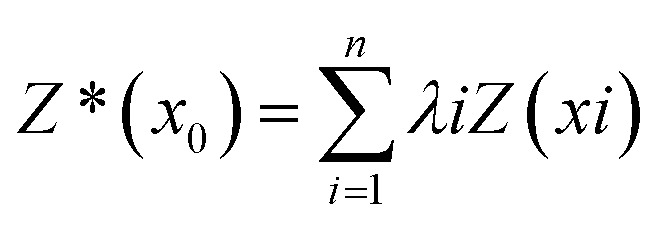


Variable descriptions are provided in the SI.

### 2.6 Source apportionment

Source apportionment was conducted using EPA PMF v5.0 following the US EPA^14^ guidelines. Data pre-processing included outlier removal using histograms and interquartile ranges following the method used by Guan *et al.*^31^ The PMF model decomposes the original concentration matrix as shown in eqn (7):7
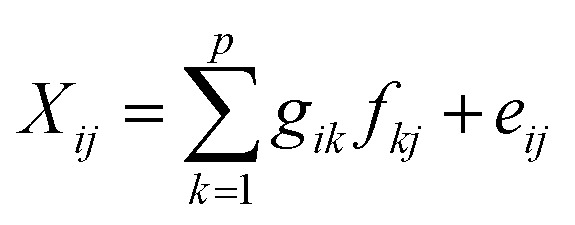


The model iteratively minimizes an objective function ‘*Q*’ to ensure the best fit, as given by eqn (8):8
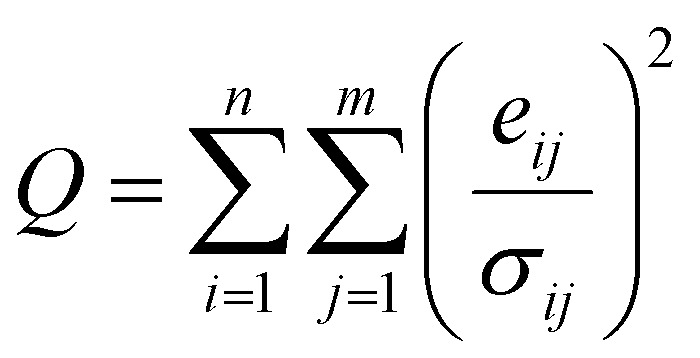


To robustly handle data variability, measurement uncertainties (Unc_*ij*_) were calculated. For concentrations above the method detection limit (MDL), uncertainty was derived from the analytical and methodological error. When data exceed or fall below the detection limits, uncertainty (Unc_*ij*_) is estimated using eqn (12) and eqn (13):9
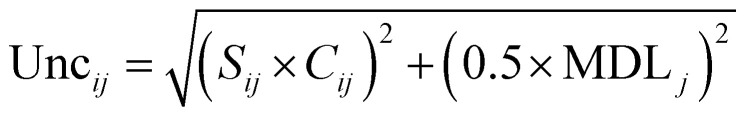
10
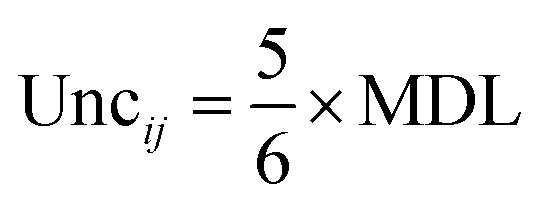


The normalized factor contribution (*g*_*ik*_) was displayed using ordinary kriging to generate continuous surfaces representing the spatial influence of each source factor across the study area. Variable descriptions are provided in the SI.

### 2.7 Machine learning-based prediction of potentially toxic element concentrations

Machine learning model development involved evaluating algorithms including feed-forward neural networks (FNN), convolutional neural networks (CNN), and random forest (RF). The deep learning models (FNN and CNN) showed lower predictive accuracy and higher variability, likely due to the limited sample size and absence of strong spatial and sequential structure in the input data. In contrast, RF demonstrated superior accuracy and a more stable performance, reflecting its robustness for handling heterogeneous environmental datasets.^32^

Accordingly, a supervised machine learning framework based on RF regression was developed to predict the PTE concentrations in soils. The modelling objective was to quantify nonlinear relationships between measured PTE concentrations and an integrated set of environmental predictors while evaluating their relative influence. The dataset for this study consisted of 120 georeferenced soil samples characterized by their physicochemical properties (pH, EC, C%, N%, CEC, and OM), spatial variables (geographic coordinates), categorical variables (soil texture, depth class, and land-use category), and factor contributions (F1–F4) obtained from the corresponding PMF *G*-matrix. To prepare the data for modelling, categorical predictors were converted into dummy variables using one-hot encoding, with one reference category omitted to prevent perfect multicollinearity. Continuous variables were standardized using *z*-score normalization. The target PTE concentrations were winsorized at the 5th and 95th percentiles to reduce skewness and limit the influence of extreme outliers.

The RF model was implemented using the TreeBagger algorithm with an ensemble of 300 bootstrap-aggregated decision trees. The model built each tree using a different bootstrap sample from the training dataset. At each split, it evaluated only a random subset of predictors, which reduces the correlation among trees and improves the overall model robustness.^20,33^ The dataset was partitioned using a 70/30 random hold-out split for model training and independent testing, respectively. Although random train–test splitting may slightly overestimate the model performance due to the spatial dependence, this is consistent with the interpolative objective of this study and was considered when interpreting the model results. The final prediction for input vector *x*_t_ was computed as the average output of all individual trees using eqn (11):11
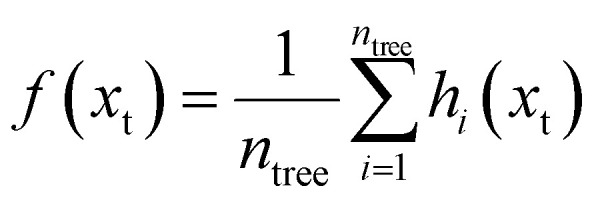
where *f*(*x*_t_) represents the final predicted output for input vector *x*_t_, *n*_tree_ denotes the total number of decision trees in the ensemble, and *h*_*i*_(*x*_t_) is the prediction made by the *i*th decision tree.

The model performance was rigorously assessed on the test set using the coefficient of determination (*R*^2^), the predictive squared correlation coefficient (*Q*^2^) and the root mean square error (RMSE) (eqn (12)–(14), respectively), where higher *R*^2^ and *Q*^2^ values and lower RMSE indicate superior predictive accuracy.12
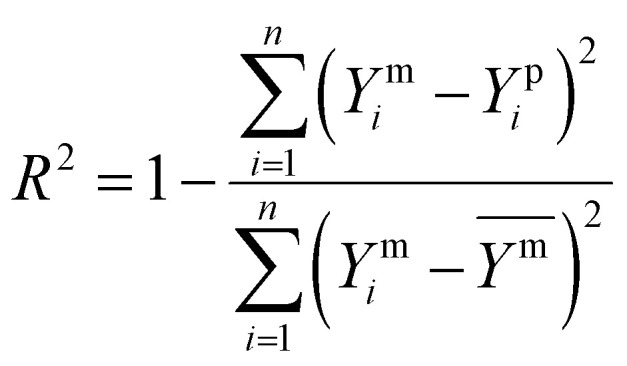
13
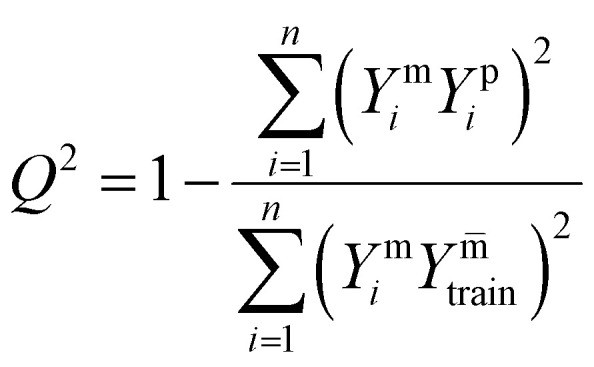
14
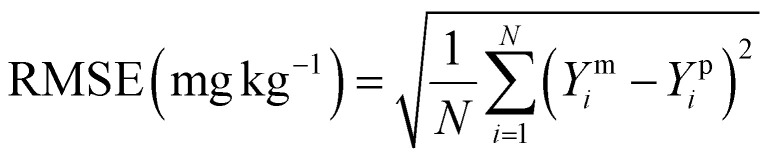
Here, *Y*^m^_*i*_ and *Y*^p^_*i*_ represent the measured and predicted values for the *i*th sample, respectively, 
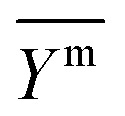
 denotes the mean of the measured values, Y^m̄^_train_ is the mean of the measured values in the training dataset and *N* is the total number of samples used in the analysis. To interpret the model, the relative feature importance of each predictor was quantified using permutation-based out-of-bag (OOB) metrics. Furthermore, the partial dependence plots were generated for variables to visualize their marginal effect on the predicted PTE concentrations, thereby fulfilling the dual objective of accurate prediction and mechanistic insight.^34^

### 2.8 Human health risk assessment

Human health risks associated with PTE exposure were evaluated following the US EPA Human Health Risk Assessment framework.^35^ The assessment quantified both non-carcinogenic (NCR) and carcinogenic risks (CR) for two sensitive sub-populations: children (1–17 years) and adults (18–65 years). PTE exposure was calculated across three pathways: ingestion, inhalation, and dermal contact. The average daily dose (ADD) for each pathway was determined using standard eqn (15) and (16). The non-carcinogenic risk was evaluated using the hazard quotient (HQ) and hazard index (HI), where HI > 1 indicates potential adverse health effects. CR for each metal was calculated using its respective slope factor, and the total cancer risk (TCR) was obtained by summing the individual CR values of all elements.15
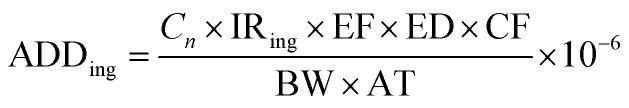
16
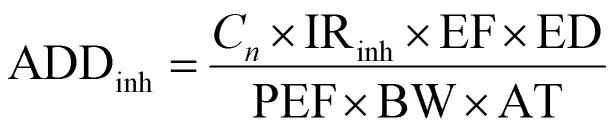
17
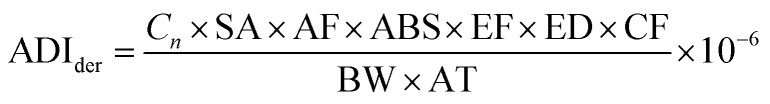
18
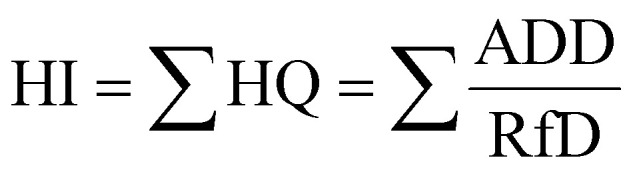

19 TCR = ∑CR = ADD × SF

Evaluation of HQ, HI, CR, and TCR was performed individually for each land use category to delineate the risk patterns across the study area. Furthermore, to better capture exposure variability and reduce uncertainty, a probabilistic risk assessment was conducted using a Monte Carlo simulation with 10 000 iterations at the 95% confidence level.^36^ For this simulation, the average measured concentrations of each PTE across the entire study area were used as the central input value, while other exposure parameters were defined by the probability distributions adopted from the established literature (detailed in Tables S2–S5). This approach provided a robust probabilistic range of risks, complementing the deterministic estimates.

### 2.9 Data analysis

The initial assessment of the PTE concentrations involved the Shapiro–Wilk test using the PAST4.17 software to evaluate distribution normality. The results indicated statistically significant deviations from a normal distribution for all analyzed elements (*p* < 0.05). Therefore, the relationships among non-normally distributed PTE concentrations were examined using Spearman's rank correlation coefficient (*ρ*). Data visualization through box plots was performed using OriginPro 2024 (10.1.0.178). Descriptive statistics (mean, mode, SD, CV%, and kurtosis) were calculated using Microsoft Excel 2023 (Microsoft, USA). The spatial distribution of PTEs and pollution indices was modelled and visualized through kriging interpolation in ArcGIS (ArcMap 10.8). For advanced modelling, RF regression and a probabilistic Monte Carlo simulation for health risk assessment were conducted using MATLAB R2025b.

## 3. Results

### 3.1 Soil physicochemical properties

The soil physicochemical properties demonstrated significant variation across land uses. A textural gradient was distinct, with CM soils identified as sandy loam, while RD, AG, FT, and RS soils were predominantly sandy clay loam. The soil pH ranged from acidic to alkaline (Table 1). CM soils were moderately acidic (mean pH: 5.8), contrasting clearly with the alkaline RD soils (8.0). AG (6.1) and FS (6.4) soils were near-neutral, while RS soils were more acidic (5.4). EC showed distinct spatial patterns, with the highest salinity in RS soils (mean: 487.4 µS cm^−1^), followed by RD (394.8 µS cm^−1^). AG and CM soils displayed lower EC (221.7 and 198.2 µS cm^−1^, respectively). Soil fertility indicators revealed a clear degradation gradient. CM soils had the lowest total carbon (0.2%), nitrogen (0.0%), and CEC (7.8 cmol(+) kg^−1^) among the land uses. In contrast, FS soils exhibited the highest fertility, with a CEC of 14.0 cmol(+) kg^−1^ and the highest carbon content (1.4%), a characteristic supported by their finer texture and undisturbed status.

**Table 1:** Descriptive statistics of the physicochemical properties of various soils^a^

	pH	EC (µS cm^−1^)	C (%)	N (%)	OM (%)	CEC (cmol(+) kg^−1^)
**Coal mine**						
Mean	5.8	198.2	0.2	0.0	0.4	7.8
MIN	4.2	56.0	0.1	0.0	0.2	7.1
MAX	6.8	643.6	0.6	0.1	0.9	10.6
SD	0.7	139.8	0.1	0.0	0.1	1.0
CV%	11.8	70.6	47.9	87.9	35.0	12.8
Kurtosis	0.3	4.2	3.0	4.1	10.4	3.6
Median	5.9	179.5	0.2	0.0	0.4	7.5

**Residential area**						
Mean	8.0	394.8	0.5	0.0	0.8	10.3
MIN	7.2	135.5	0.4	0.0	0.6	9.9
MAX	8.3	822.9	0.6	0.1	1.0	10.8
SD	0.3	171.5	0.0	0.0	0.1	0.2
CV%	4.0	43.4	9.8	14.2	9.8	2.4
Kurtosis	0.6	0.5	0.0	2.6	0.1	−0.2
Median	8.0	367.2	0.5	0.0	0.8	10.2

**Agricultural area**						
Mean	6.1	221.7	0.8	0.1	1.5	12.1
MIN	5.8	39.0	0.5	0.0	1.4	9.6
MAX	6.6	510.0	1.0	0.1	1.7	13.1
SD	0.2	105.3	0.1	0.0	0.1	1.0
CV%	3.2	47.5	18.1	22.5	5.3	8.3
Kurtosis	0.0	1.8	0.8	0.8	−0.2	1.8
Median	6.1	210.6	0.9	0.1	1.5	12.4

**Forest area**						
Mean	6.4	350.0	1.4	0.1	2.8	14.0
MIN	6.1	265.5	0.8	0.1	1.4	11.8
MAX	6.9	413.3	2.0	0.2	3.5	15.9
SD	0.3	47.3	0.5	0.0	0.8	1.4
CV%	3.9	13.5	35.1	30.1	26.8	10.2
Kurtosis	−0.7	−1.1	−2.0	−1.9	−0.4	−1.8
Median	6.3	350.2	298.7	1.7	3.1	0.2

**Roadside area**						
Mean	5.4	487.4	0.7	0.1	0.9	10.1
MIN	4.6	300.7	0.3	0.0	0.5	8.1
MAX	6.5	680.3	2.1	0.2	3.1	16.0
SD	0.6	112.0	0.7	0.1	0.9	3.0
CV%	10.7	23.0	92.5	98.0	92.8	30.0
Kurtosis	−0.8	−1.0	−0.6	−0.7	3.1	−0.6
Median	5.3	480.8	0.4	0.0	0.6	8.5
BV	5.4	487.4	0.7	0.1	0.9	10.1

a Background value (BV)

### 3.2 Potentially toxic element concentration

The PTE concentrations demonstrated significant enrichment relative to the background values (BV) across all land uses, with CM soils exhibiting the most severe contamination (Table 2). In CM soils, the mean concentrations of Zn (266.4 mg kg^−1^), Cd (3.2 mg kg^−1^), and Hg (1.0 mg kg^−1^) exceeded their respective BVs by approximately 4.7, 3.4, and 2.0 times, respectively, while the Zn concentrations were more than one order of magnitude higher than the Indian natural background values (INBV). Co also showed pronounced enrichment, with concentrations in CM and AG soils exceeding both the local BVs and INBV. The pattern of elevated Zn, Pb, and Cd extended to other land uses, with AG soils showing the highest Cd enrichment. The Cd levels across the studied land uses were 3.5- to 9.7-times higher, the Zn levels were 2.8- to 8.5-times higher, and the Pb levels were 2.0- to 4.5-times higher than the averages reported from other Indian mining areas (Table 3). When compared against international soil quality guidelines, the Cd concentrations across all land uses surpassed the Canadian Council of Ministers of the Environment (CCME) guidelines, the Zn concentrations exceeded the CCME limit of 200 mg kg^−1^ in CM and AG soils; and both the Ni and Cr concentrations exceeded their respective CCME thresholds (50 mg kg^−1^ for Ni and 64 mg kg^−1^ for Cr) across all land uses, whereas Hg, As, Pb, and Cu remained below the CCME guideline values.

**Table 2:** Descriptive statistics of the PTE concentrations (mg kg^−1^) of various soils^a^

	Hg	As	Zn	Pb	Co	Cd	Ni	Cr	Cu
**Coal mine**									
Mean	1.0	15.6	266.4	59.0	172.4	3.2	395.5	333.4	236.2
MIN	0.4	10.2	201.0	36.0	120.5	1.2	249.8	102.5	92.5
MAX	2.0	20.9	337.7	76.0	274.8	4.6	491.0	391.1	370.1
SD	0.5	3.8	41.5	9.0	45.8	0.9	90.3	98.0	5.0
CV%	46.3	24.1	15.6	15.2	26.6	27.7	22.8	42.0	22.0
Kurtosis	−0.3	−1.5	−1.2	1.2	0.4	−0.3	−1.4	−1.8	−0.8
Median	0.8	15.3	274.1	59.7	151.9	3.5	424.1	260.1	271.3

**Residential area**									
Mean	0.7	2.9	174.7	27.4	79.6	3.1	234.1	152.0	86.0
MIN	0.1	0.9	78.8	17.0	49.7	1.0	141.2	103.6	43.7
MAX	2.0	6.1	331.0	37.9	123.5	6.8	374.4	188.0	152.0
SD	0.15	1.4	65.0	6.2	18.0	1.5	70.6	28.1	27.3
CV%	73.3	47.4	37.2	22.8	22.7	50.1	30.2	18.5	31.8
Kurtosis	−0.4	−0.2	0.3	−1.1	0.6	0.1	−0.3	−1.0	1.6
Median	0.8	3.1	159.0	27.5	83.4	3.2	233.2	166.2	87.3

**Agricultural area**									
Mean	1.5	8.9	260.6	16.7	160.9	10.7	325.6	181.7	179.1
MIN	0.1	1.9	204.2	12.1	109.8	5.0	246.1	113.0	111.3
MAX	2.8	12.3	300.9	19.5	190.6	14.5	360.8	275.6	275.6
SD	0.7	2.3	24.2	2.2	22.4	2.6	32.4	35.7	43.9
CV%	47.5	25.6	9.3	13.0	13.9	24.4	10.0	19.7	24.5
Kurtosis	−0.5	3.2	0.1	−0.4	0.6	0.2	1.3	1.9	0.4
Median	1.4	9.1	260.8	17.1	165.5	12.3	330.8	180.3	180.7

**Forest area**									
Mean	0.2	9.1	146.0	40.2	118.1	2.6	248.9	117.9	96.2
MIN	0.0	2.2	98.5	24.4	89.5	0.5	190.5	95.5	66.9
MAX	0.7	13.1	190.3	57.9	182.3	5.5	315.7	142.3	106.6
SD	0.2	3.3	34.5	12.7	25.5	1.6	38.8	14.8	8.2
CV%	121.7	36.3	23.6	31.6	21.6	62.0	15.6	12.5	8.5
Kurtosis	1.8	−1.0	−1.7	−1.8	2.0	−1.1	−1.2	−1.3	8.2
Median	0.1	10.7	147.9	42.9	110.2	2.5	250.5	112.3	98.2

**Roadside area**									
Mean	0.8	4.9	165.5	56.7	141.4	5.8	255.6	99.7	119.3
MIN	0.2	1.3	100.7	45.7	110.9	1.2	120.2	85.7	94.3
MAX	1.5	8.9	210.2	65.8	160.2	9.5	332.8	126.6	144.7
SD	0.4	2.4	32.9	6.2	12.6	2.4	42.8	11.3	16.5
CV%	46.8	49.7	19.9	10.9	8.9	41.2	16.8	11.3	13.9
Kurtosis	−0.6	−1.1	−0.2	−1.0	0.7	−0.6	6.5	0.5	−1.2
Median	0.8	4.2	170.3	55.9	140.6	6.1	260.9	100.1	115.9
BV	0.5	3.4	56.9	66.6	23.2	0.95	67.8	169.9	73.6
INBV	—	—	22.1	13.1	15.2	—	27.7	114	56.5
ILS	0.2	5	250	5	—	1	20	5	25
WHO	—	—	50	85	—	0.8	35	100	36
CCME	6.6	12	200	70	—	1.4	50	64	91

a Background value (BV), Indian natural background value (INBV),^37,38^ Indian limits of soil (ILS),^38^ World Health Organization (WHO),^39^ Canadian Council of Ministers of the Environment (CCME).^40^

**Table 3:** Potentially toxic element concentrations in the soils of coal mining regions globally (mg kg^−1^)

Coal mine site, country	Land uses	Potentially toxic element (mg kg^−1^)									Reference
		Hg	As	Zn	Pb	Co	Cd	Ni	Cr	Cu	
Coal-mine brownfield, Beijing, China	NA	NA	NA	87.29	55.35	NA	0.29	30.98	48.56	39.26	11
Shandong province, China	Farmland	NA	NA	66.30	23.71	NA	0.14	29.53	72.16	23.07	53
Jharia CF, Jharkhand, India	Roadside soil	0.43	3.55	5.49	1.2	1.63	1.68	NA	2.34	2.06	54
Enugu coal mine, Nigeria	Abandoned coal mine	NA	58.83	353.34	121.39	NA	3.34	40.32	137.12	68.19	12
Coal mine soils, China	Coal mine	0.09	29.28	79.40	41.01	NA	0.51	47.10	92.89	33.18	6
Yunnan (China)	Coal mine	NA	NA	2273.77	1117.4	NA	NA	110.59	148.27	191.05	55
Open-cast coal mines, Raniganj basin, India	NA	NA	9.56	54.60	122.7	25.5	0.92	43.3	265.4	42.7	9
Coal mines soil, eastern India	NA	NA	NA	19.90	11.43	6.86	0.80	11.38	23.40	11.36	56
Coal mines in various cities/countries	NA	0.02–0.69	0.5–38.3	1.5–296	0.5–433	NA	0.02–4.48	4.3–390	17.5–954	0.5–110	10
JVR-OC, India	Coal mine	1.0	15.6	266.4	59.0	172.4	3.2	395.5	233.4	233.4	Current study
	Residential area	0.7	2.9	174.7	27.4	79.6	3.1	234.1	152.0	86.0	
	Agricultural area	1.5	8.9	260.6	16.7	160.9	10.7	325.6	181.7	179.1	
	Forest area	0.2	9.1	146.0	40.2	118.1	2.6	248.9	117.9	96.2	
	Roadside	0.8	4.9	165.5	56.7	141.4	5.8	255.6	99.7	119.3	

### 3.3 Machine learning-based prediction of potentially toxic element concentrations

**Fig. 2:**
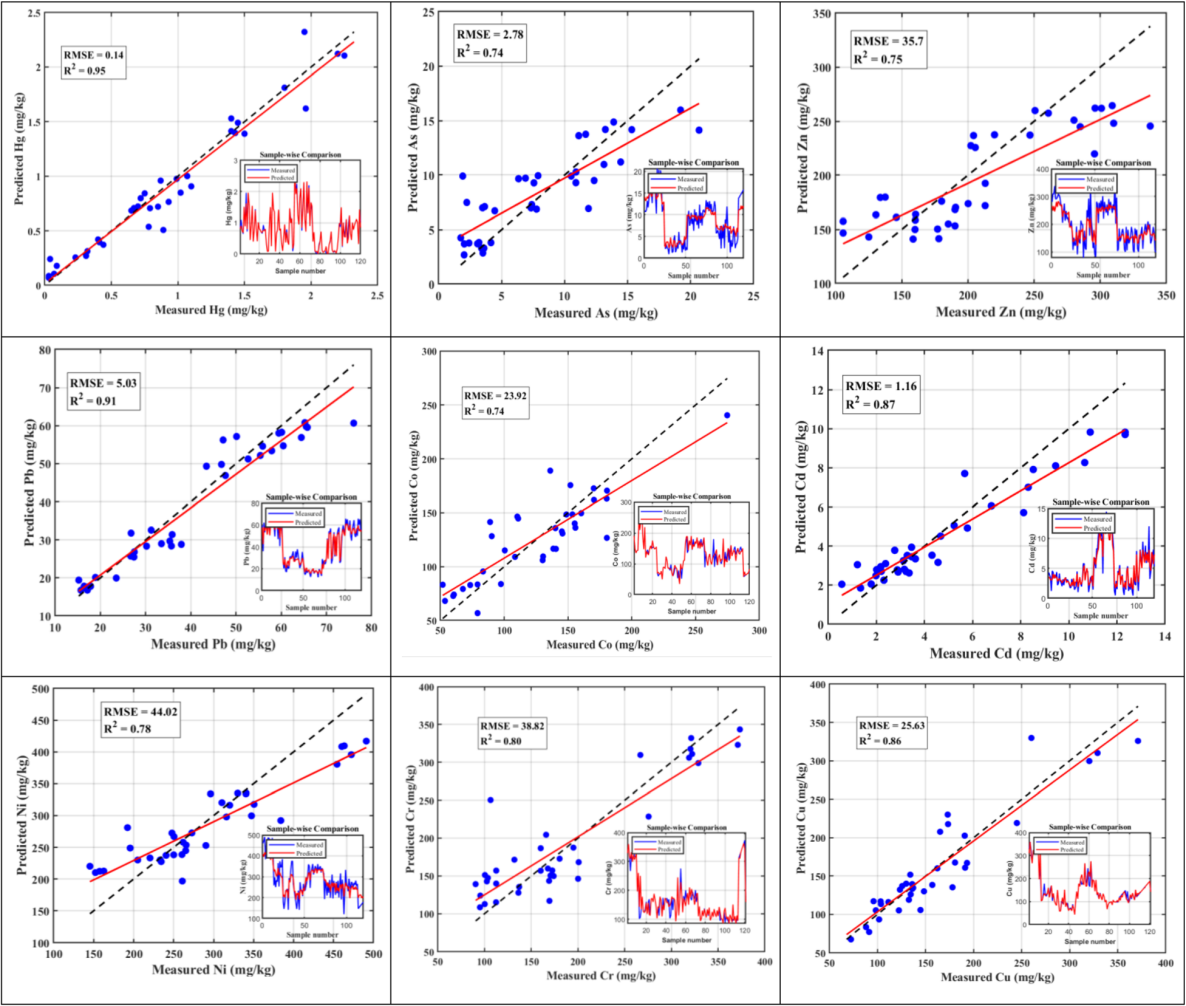
RF model performance for predicting the concentration of potentially toxic elements, showing scatter plots of measured *versus* predicted values with *R*^2^ and RMSE (mg kg^−1^) metrics. The red solid line represents the linear regression fit, while the black dashed line denotes the 1 : 1 reference line.

The predictive performance of the RF model was the highest for Hg (*R*^2^ = 0.95), followed by Pb (*R*^2^ = 0.91), Cd (*R*^2^ = 0.87), and Cu (*R*^2^ = 0.86) (Fig. 2). Cr (*R*^2^ = 0.80), Ni (*R*^2^ = 0.78), and Zn (*R*^2^ = 0.75) showed moderate predictive accuracy, while As and Co exhibited the lowest *R*^2^ values (both 0.74). The predictive accuracy reflected by the RMSE values ranged from 0.14 mg kg^−1^ for Hg to 44.02 mg kg^−1^ for Ni, with low errors for Cd (1.16 mg kg^−1^) and Pb (5.03 mg kg^−1^), and higher errors for Cu (25.6 mg kg^−1^), Co (23.92 mg kg^−1^), Zn (35.7 mg kg^−1^), and Cr (38.38 mg kg^−1^). The cross-validation results indicated *Q*^2^ values of 0.95 for Hg and 0.92 for Pb, decreasing to 0.70 for Ni, with intermediate *Q*^2^ values of 0.87 for Cu, 0.82 for Cd, 0.81 for Cr, 0.74 for Co, 0.73 for As, and 0.59 for Zn (Table S8).

The feature importance analysis identified distinct environmental drivers for each element (Fig. S6). As was mainly influenced by land use (RD) and soil pH, while Cd was strongly linked to PMF Factor 4. The Co model was primarily driven by RD land use and PMF Factor 1, while Cr concentrations were best explained by PMF Factor 2 and EC. Cu variability was largely controlled by PMF Factor 2 and soil pH, whereas Zn was predominantly influenced by AG land use together with PMF Factors 4 and 2. Pb exhibited a strong dependence on a PMF-derived source and CEC, Hg was mainly governed by PMF Factors 3 and 1, and Ni was chiefly associated with PMF Factors 1 and 2. Additionally, the OOB error analysis for all PTEs demonstrated model robustness, with errors stabilizing as the number of trees increased, confirming that the ensemble size was sufficient for reliable predictions.

### 3.4 Spatial distribution of potentially toxic element contamination

**Fig. 3:**
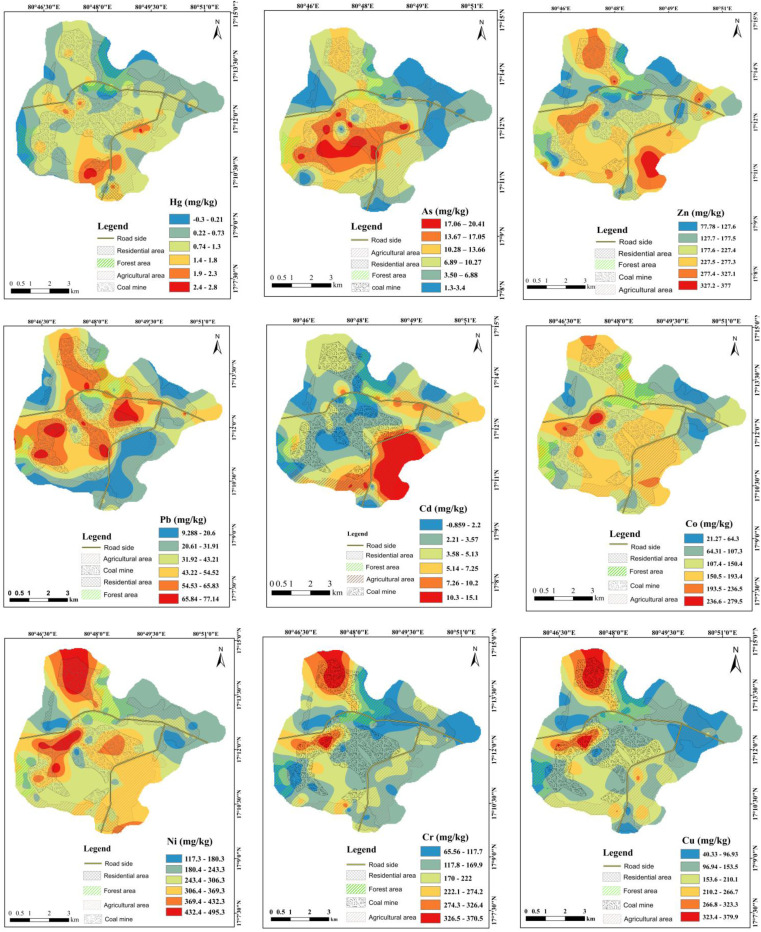
Spatial distribution patterns of potentially toxic elements in different land use types.

Kriging interpolation revealed heterogeneous yet distinct patterns of PTE distribution, defining clear contamination hotspots mainly influenced by proximity to the coal mine (Fig. 3). A concentration gradient was evident for several metals, with the highest concentrations clustered within or immediately adjacent to the mining zone. Cr and Cu exhibited strong clustering in the central-south-western and northern CM zones, while Ni and Pb formed hotspots in the central and northern CM with Pb hotspots further extending into the RS and FT zones. In contrast, Zn showed broad dispersion across the central CM and hotspots in the eastern AG areas. As and Co shared a similar pattern of enrichment in the central, northern and western CM zones. Hg and Cd demonstrated more localized, point-source-like enrichment, with Hg hotspots in the southern CM and Cd accumulation in the AG and RD zones.

### 3.5 Environmental risks assessment

#### 3.5.1 Pollution risk

**Fig. 4:**
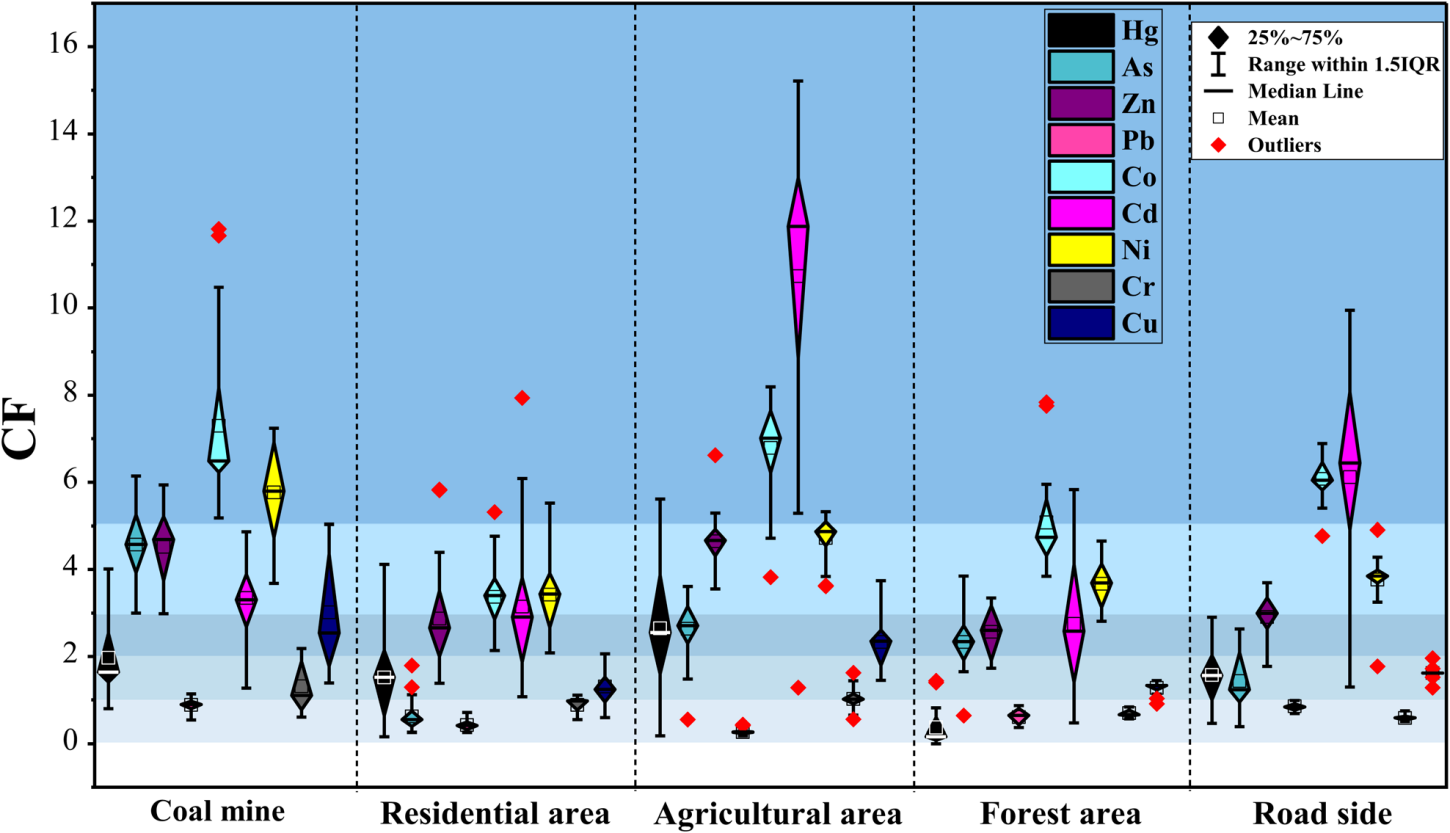
Contamination factor (CF) of potentially toxic elements across different land use types.

The CF analysis revealed distinct contamination patterns across land-use types (Fig. 4). CM soils were characterized by consistently severe Co contamination, with 100% of samples exhibiting CF > 5, identifying Co as the dominant contaminant in this land use. Ni also showed uniformly high CF values. In addition, Zn frequently reached severe contamination levels, with approximately 37.5% of samples showing CF > 5. Cr exhibited low CF values (<2) throughout the CM sites, while Cd and Pb generally remained within the moderate contamination range. AG soils showed severe Cd contamination, with 100% of samples exhibiting CF > 5. Co and Ni also displayed elevated CF values, with many samples falling within the severe contamination category. Zn frequently approached or exhibited CF > 5, whereas Cr remained at low to moderate levels across AG sites. RD soils were mainly characterized by moderate to considerable contamination, with Co and Ni showing higher CF values relative to other elements. Cr remained consistently low, while Cd showed localized enrichment. FT soils exhibited moderate Co and Ni contamination, with occasional samples reaching CF > 5. The Cr values were consistently low (<1), and other metals generally remained within the moderate contamination range. RS soils showed considerable to severe Cd contamination, with multiple samples exhibiting CF > 5. Co and Ni were also elevated, with several samples approaching or exceeding the severe pollution threshold. Zn occasionally exhibited high CF values, whereas Cr remained low to moderate.

**Fig. 5:**
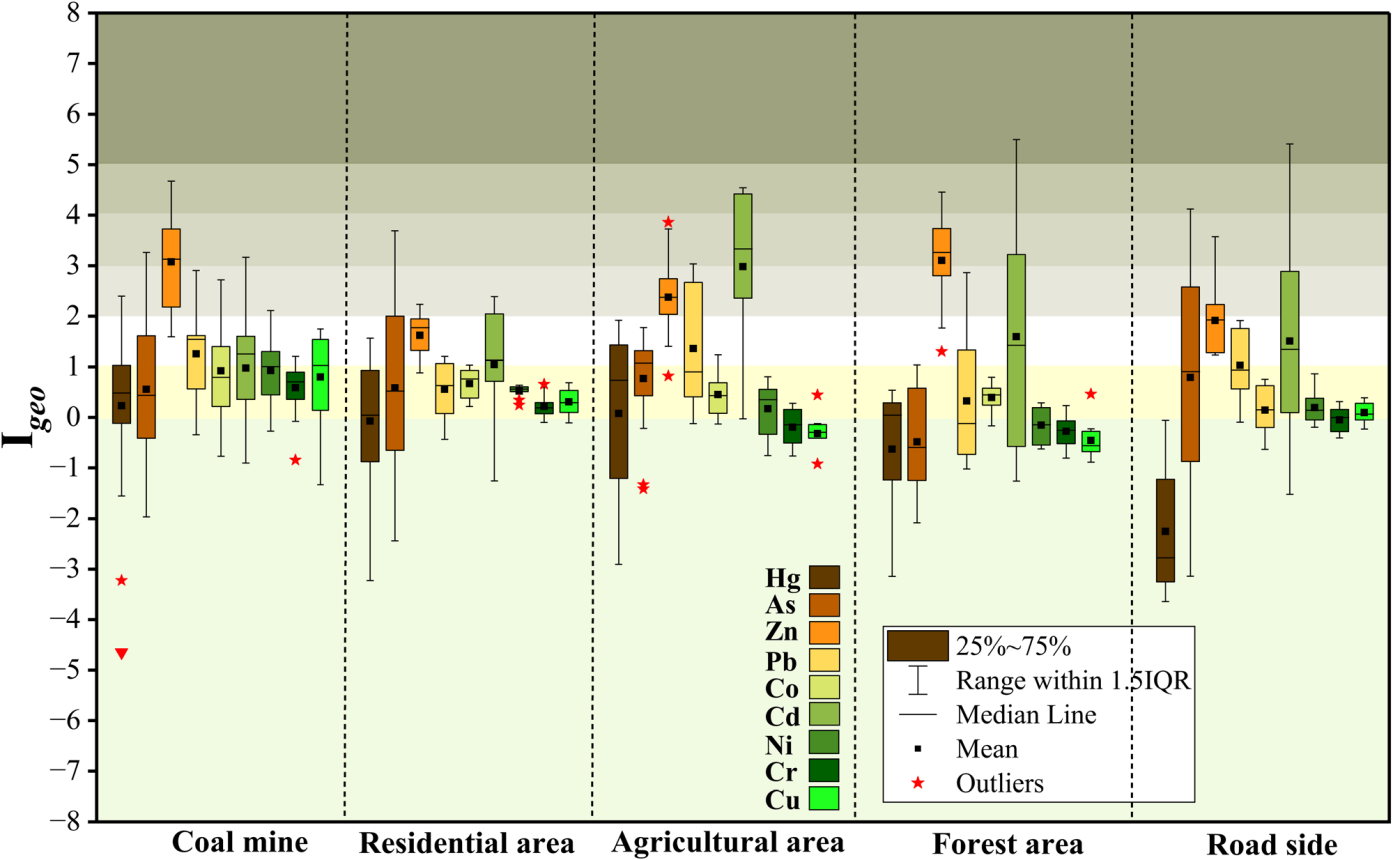
*I*_geo_ values of potentially toxic elements across different land use types.

The *I*_geo_ confirmed widespread moderate to heavy soil contamination, with the specific metals and severity varying between land uses (Fig. 5). The CM area was the most affected, with 53.65% of samples moderately to heavily contaminated with Zn, alongside significant contributions from Pb, Co, and Cd (9.7%, 9.7%, and 7.3%, respectively). This was followed by AG soils, where Cd was the dominant contaminant (55.5% of samples), with substantial Pb (33.3%) and Zn (22.2%) contamination. FT and RS zones also showed considerable pollution, each with 40–50% of samples contaminated by Zn and Cd. RD soils exhibited lower but notable contamination, primarily from Cd (28.57%) and Zn (14.28%).

**Fig. 6:**
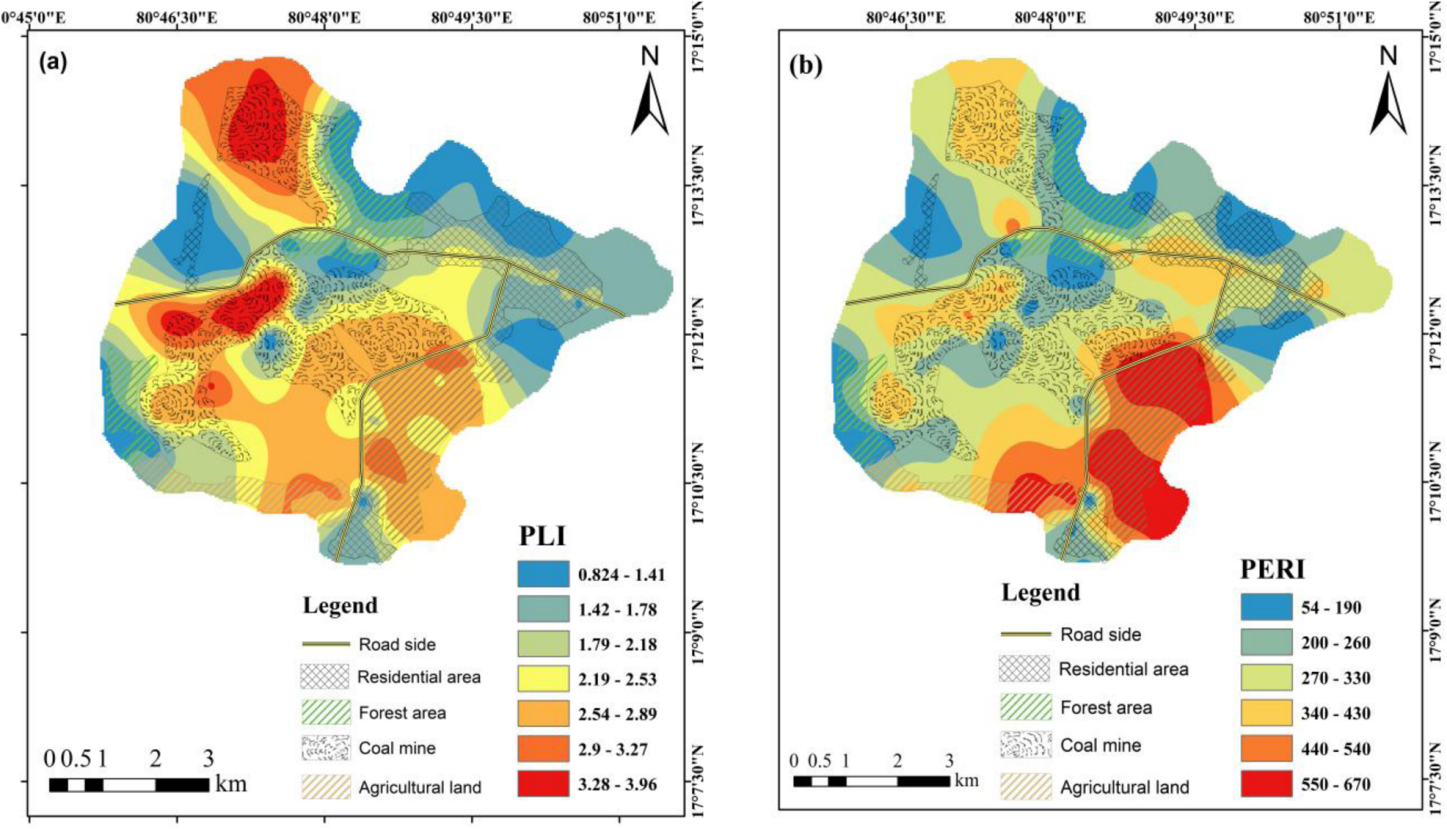
Spatial distribution of (a) pollution load index (PLI) and (b) potential ecological risk index (PERI) across the study area.

The PLI corroborated these findings, with mean values decreasing in the order of CM (2.8) > AG (2.5) > FT (1.98) > RD (1.79) > RS (1.59), confirming the CM and AG areas as pronounced pollution hotspots (Fig. 6a). Collectively, the indices consistently identified Zn, Cd, and Pb as the principal contaminants across the study area.

#### 3.5.2 Ecological risk

**Fig. 7:**
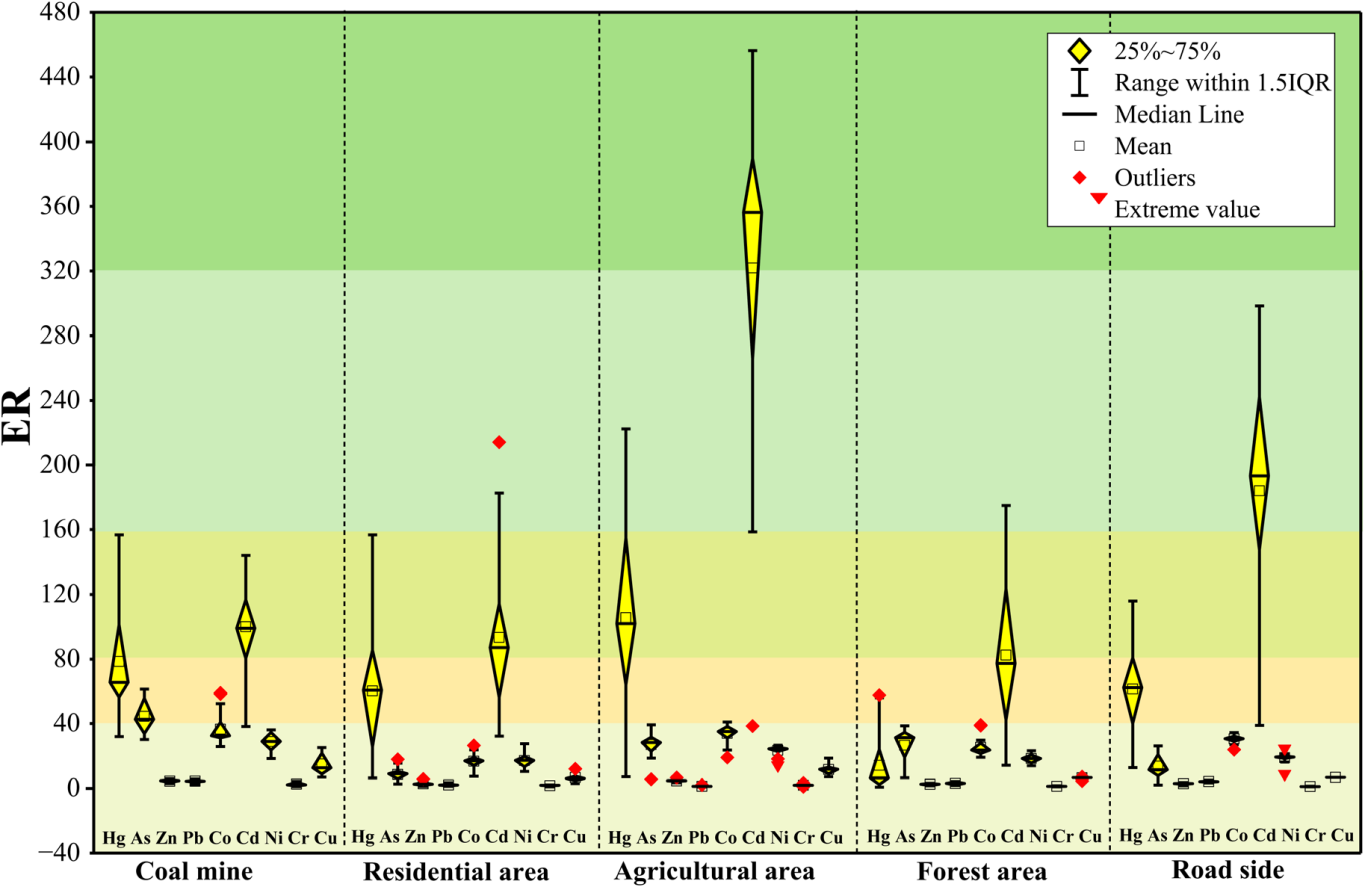
Ecological risk (ER) values of potentially toxic elements across different land use types.

The ER assessment identified Cd as the principal contributor to ecological risk, reaching high to very high-risk levels (ER ≥ 160 and ≥320) mainly in the AG and RS soils (Fig. 7). Hg generally posed moderate to considerable ecological risk, with occasional high-risk values in the AG and RS areas. In contrast, Zn, Pb, Ni, Cr, and Cu were largely associated with low to moderate ecological risk across all land uses. The spatial distribution of PERI values showed clear land-use-dependent patterns (Fig. 6b). AG soils exhibited the highest ER, with most sites falling in the considerable (300–600) category and several exceeding ≥600 (high risk). The CM and RS areas were dominated by considerable ER (300–600), with a smaller number of sites reaching the high-risk category. RD soils were mainly characterized by moderate (150–300) to considerable risk, whereas FT soils largely fell within the low (<150) to moderate (150–300) risk categories, indicating comparatively lower cumulative ecological risk.

### 3.6 Source apportionment of potentially toxic elements

**Fig. 8:**
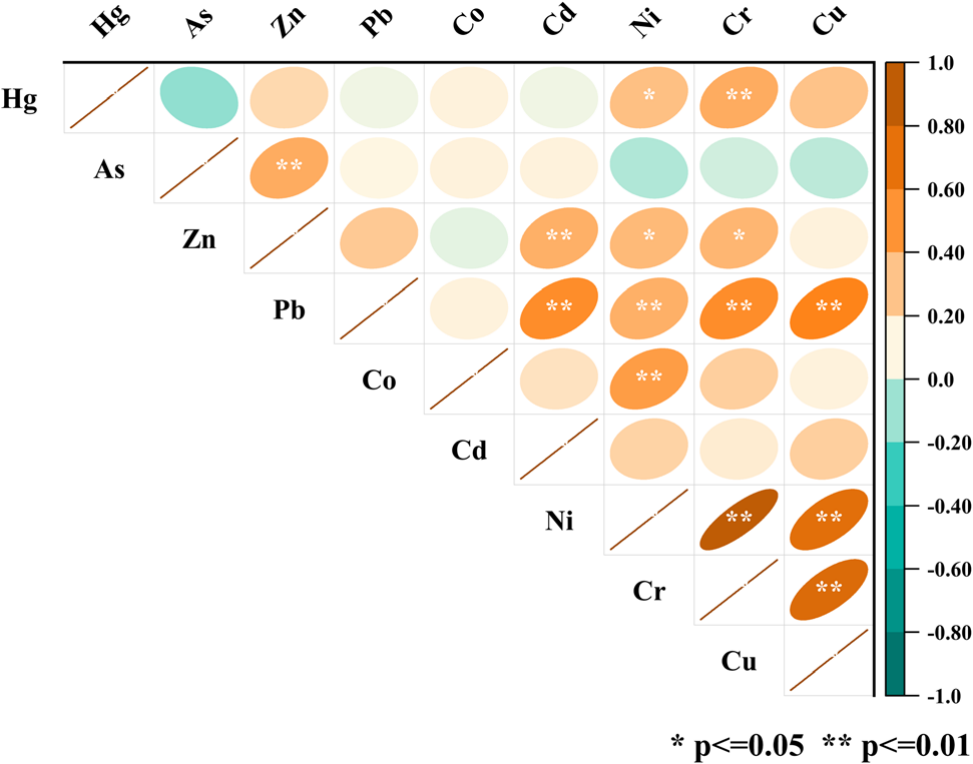
Spearman's correlation matrix of potentially toxic elements with statistically significant relationships indicated at *p* ≤ 0.05 and *p* ≤ 0.01.

The soil PTEs in the study area exhibited non-normal distributions, as confirmed by Shapiro–Wilk tests (*p* < 0.05). Subsequent Spearman's correlation analysis revealed significant positive relationships among several elements, including strong correlations for Ni–Cr (*ρ* = 0.81), Cr–Cu (*ρ* = 0.68), and Ni–Cu (*ρ* = 0.62) (Fig. 8). Moderate correlations were found for pairs such as Pb–Cu and Cd–Pb, while weaker or negative associations were noted for Hg–As (*ρ* = −0.20) and As–Ni (*ρ* = −0.14).

A robust four-factor PMF solution was identified as optimal after testing 3–5 factors based on the lowest *Q* value and stable residuals. Model validation confirmed its reliability as most elements were classified as “strong” (*r*^2^ = 0.63–0.99), with As being the sole “weak” variable (*r*^2^ = 0.5) (Fig. S2). Uncertainty analyses using the DISP method demonstrated strong stability (<1% reduction in Q), and the bootstrap results showed that all factors were unmixed and matched the base case in over 85% of runs.^41,42^ The BS-DISP analysis further identified Pb and Hg as the most sensitive elements, each showing 80% sensitivity in Factor 1 and Factor 3, respectively, while Zn, Cr, and Cu also exhibited significant influence (>50%) as key tracers (Fig. S4, S5a and b).

**Fig. 9:**
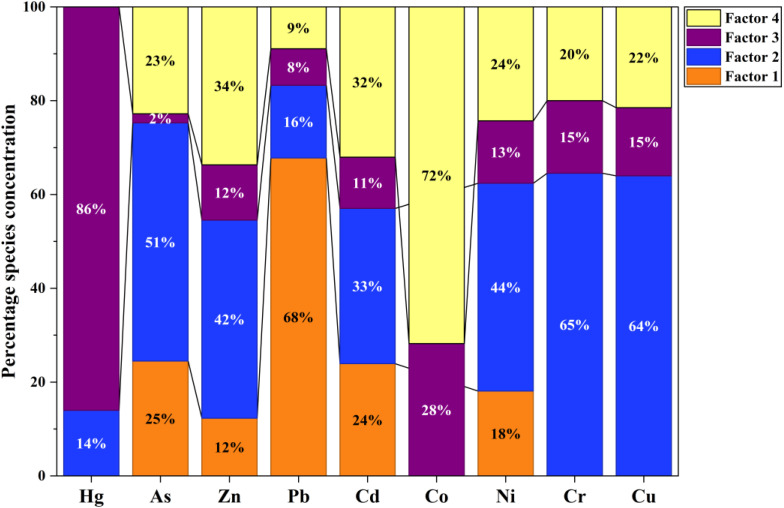
Percentage contributions of individual PMF factors towards each potentially toxic element.

The four-factor PMF solution resolved distinct source contributions, with Factor 2 contributing the largest share of the PTE (36.51%), followed by Factor 4 (26.11%), Factor 3 (21.10%), and Factor 1 (16.29%) (Fig. 9, S3 and S1). In terms of elemental contributions, Factor 1 was dominated by Pb (67.8%), with additional contributions to Cd (23.9%), Co (28.5%), and Ni (18.1%). Factor 2 showed the highest contributions to As (50.8%), Zn (42.3%), Ni (44.3%), Cr (64.6%), and Cu (64.0%), indicating its dominant influence across multiple elements. Factor 3 contributed modestly, mainly to Hg (14.0%), Zn (11.8%), Cd (10.9%), and Ni (13.3%). In contrast, Factor 4 was strongly associated with Hg (86.0%) and Co (71.8%) and showed substantial contributions to Cd (32.0%), Zn (33.7%), Ni (24.3%), Cr (20.0%), and Cu (21.5%).

**Fig. 10:**
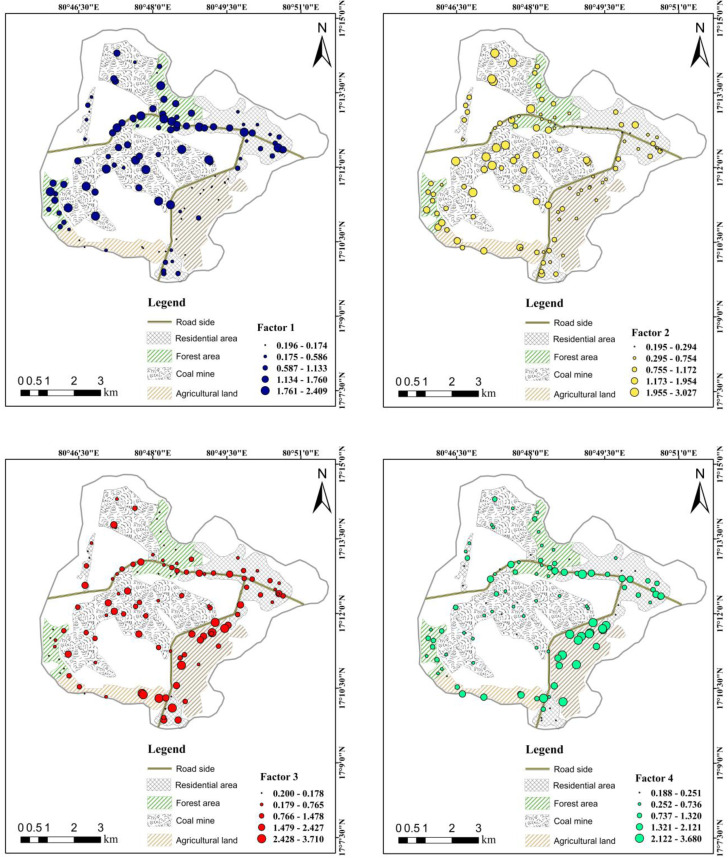
Spatial distribution of PMF factors (1–4), showing the variations in the factor contribution values across all sampling points. Variable-sizedcircles represent low to high factor loading.

The spatial interpolation of the PMF factor contributions at individual sampling sites revealed distinct geographical patterns for all four factors (Fig. 10 and S7). Factor 1 showed its highest contributions concentrated within the CM area, with moderate values extending into the RS and FT zones. Factor 2 exhibited the highest contribution levels (2.7–3.1) in the northern and central-western area, forming pronounced hotspots in the CM zones. Moderate to low values (0.19–0.74) were mainly distributed across the eastern and south-eastern area. Factor 3 displayed a heterogeneous spatial pattern, with localized hotspots (3.3–3.8) primarily in the southern sector, particularly in the AG and CM areas. Intermediate contributions (2.0–2.5 and 2.6–3.2) extended toward the central corridor, while lower values (−0.76 to 0.55) were predominantly observed in the northern and north-eastern regions, corresponding mainly to the FT and RD areas. In contrast, Factor 4 showed moderate contributions across most of the study area, with values ranging from 0.01 to 3.8. The strongest hotspots were concentrated primarily within the AG area, while zones near major RS corridors exhibited intermediate contributions (1.5–2.6). The lowest values (0.01–0.8) were mainly observed in the northern FT area and dispersed RD zone.

### 3.7 Deterministic and probabilistic human health risk assessment

Non-carcinogenic risk (HQ and HI) varied across land-use categories, with the adult HI values remaining below the threshold of concern (HI < 1) (Table S6). For adults, Cr, Co, and Pb were the major contributors to HI, with Cr showing the highest HQ values (1.94 × 10^−1^–3.38 × 10^−1^) across all land uses. In contrast children showed substantially higher susceptibility, with HI values between 2.22 (AG) and 3.10 (CM), exceeding the safe limit (HI > 1) across all land uses. Cr accounted for the largest fraction of NCR in children (9.38 × 10^−1^ to 1.63), followed by Pb and Co, indicating elevated exposure risks for younger populations, particularly in coal-mine areas (Table S7).

The CR for adults ranged from 1.29 × 10^−3^ (FT) to 2.14 × 10^−3^ (CM), remaining within the US EPA acceptable to tolerable risk range (1 × 10^−6^–1 × 10^−4^ to 1 × 10^−3^), with Ni and Cr being the dominant contributors. Children exhibited higher cumulative CR values, ranging between 3.41 × 10^−3^ (FT) and 5.74 × 10^−3^ (CM), primarily driven by Ni and As. Across all land uses, CM areas consistently showed the highest total CR and HI for both age groups, whereas the FT and AG zones recorded comparatively lower but still notable risks. Overall, children experienced substantially higher non-carcinogenic and carcinogenic risks than adults across all land-use categories.

**Fig. 11:**
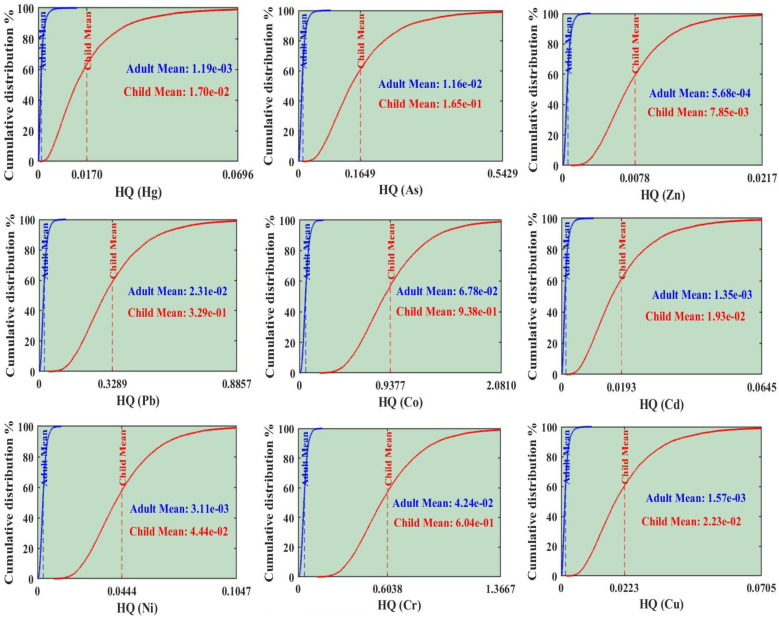
Cumulative distribution of the non-carcinogenic hazard quotient (HQ) for Hg, As, Zn, Pb, Co, Cd, Ni, Cr, and Cu, with blue and red dashed lines representing the average values for adults and children, respectively.

Based on the probabilistic health risk assessment, significant health risks were identified, with children being a particularly vulnerable population. For NCR, the HQ for both adults and children followed the order of Co > Cr > Pb > As > Ni > Cu > Cd > Hg > Zn (Fig. 11). However, HI differed critically between the two groups. The HI for adults was 1.50 × 10^−1^, which is below the safety threshold of 1, indicating no significant overall non-carcinogenic risk. In contrast, the HI for children was 2.15, exceeding the safety threshold and indicating a definite non-carcinogenic risk. Co was the dominant contributor to this NCR risk, accounting for approximately 44% of the HI for both adults and children.

**Fig. 12:**
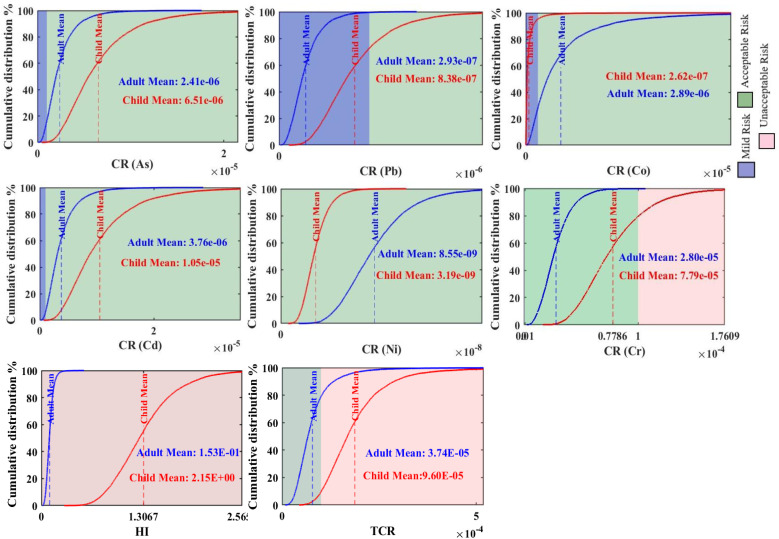
Probability of carcinogenic risk for As, Pb, Co, Cd, Ni, and Cr, with horizontal lines showing the mean values for adults (blue) and children (red), alongside the hazard index and total cancer risk for potentially toxic element concentrations.

The TCR for both adults (3.73 × 10^−5^) and children (9.60 × 10^−5^) remained within the acceptable risk range, although the values for children approached the upper threshold (Fig. 12). The ranking of PTEs by CR differed between the groups: for adults, the order was Cr > Cd > Co > As > Pb > Ni, while for children, it was Cr > Cd > As > Pb > Co > Ni. Cr was the most significant carcinogenic concern, responsible for approximately 75% of the TCR in adults and 81% in children. These results underscore that children face significantly higher health risks in the study area.

## 4. Discussion

### 4.1 Variability in physicochemical properties and potentially toxic element concentration

The severe variation in soil physicochemical properties across the study area highlights the dominant influence of anthropogenic land use on the natural soil matrix.^43,44^ The acidic conditions observed in the CM soils are a classic signature of acid mine drainage, resulting from the oxidation of sulphide minerals (*e.g.*, pyrite) released during mining operations. This degradation directly enhances the mobility and bioavailability of redox-sensitive and divalent metals such as Cd, Co, Ni, and Zn, explaining their severe enrichment in CM soils relative to the background values.^45^

In contrast, the alkaline pH in RD soils likely reflect inputs from the widespread use of construction materials and lime-rich debris, which promote metal immobilization through precipitation and adsorption mechanisms.^9^ However, despite their more favourable pH conditions, RD soils still exhibited elevated concentrations of several PTEs, indicating continued influence from atmospheric deposition and lateral transport from adjacent mining and traffic corridors. The degraded soil health in the CM areas, as evidenced by the lowest CEC, total carbon, and nitrogen, is a direct consequence of topsoil removal and organic matter depletion, severely compromising the natural resilience and capacity of the soil to retain contaminants.^46^ This land-use-driven geochemical baseline could accelerate PTE enrichment. AG soils displayed near-neutral pH but high Cd and Zn concentrations, highlighting that favourable physicochemical conditions alone do not prevent contamination when external inputs are substantial.

The CM zone emerged as the primary contamination hotspot, with Zn, Cd, Hg, Co, Ni, and Cr exceeding both the local background values and, in several cases, the Indian natural background values by multiple times. Particularly notable is the enrichment of Co and Ni, which exceeded the Indian background levels across most land uses, suggesting a strong lithogenic contribution amplified by mining disturbance and enhanced weathering of mafic and coal-bearing strata.^47^ Importantly, contamination extended beyond the mine boundary. The highest Cd concentrations were recorded in the AG soils, implicating phosphate fertilizer application and irrigation with contaminated surface water as secondary enrichment pathways.^48–50^ Similar patterns have been reported in coal mining regions of China and Nigeria;^12,51^ however, the concentrations observed here substantially exceed the average Indian mining-affected soils, underscoring the severity of cumulative anthropogenic pressure in the Godavari Valley coalfields.

The high coefficients of variation and kurtosis values further indicate strong spatial heterogeneity, consistent with the localized deposition of windborne coal dust, episodic runoff transport, and variable land management practices.^23,52^ These findings emphasize that contamination in mining landscapes is not spatially uniform and requires site-specific intervention strategies.

### 4.2 Source apportionment

The four-factor PMF solution provides a robust and geochemically interpretable separation of contamination sources. Factor 2, characterized by strong loadings of Cr, Cu, Ni, Zn, and As, represents a mixed geogenic–mining source linked to coal-bearing strata excavation and ore handling.^57^ The strong Ni–Cr and Cr–Cu correlations provide independent statistical confirmation of this factor, as these elements commonly co-occur in coal seams and associated sedimentary lithologies and are jointly mobilized during mining and material handling.^52,58,59^ These collective observations align with the findings by Zhang *et al.*^60^ and Wang *et al.*^61^ on characteristic PTE associations in mining-impacted soils. The spatial dominance of Factor 2 within the CM zones confirms direct mining influence as the principal driver of overall PTE burden.

Factor 4, dominated by Hg and Co with substantial contributions from Cd and Zn, shows strong spatial association with the AG and RS areas, indicating secondary redistribution through atmospheric deposition, fertilizer inputs, and vehicular emissions.^62,63^ The moderate positive correlations between Cd–Zn support a shared anthropogenic influence, consistent with fertilizer application, atmospheric deposition, and traffic-related inputs.^64,65^ The spatial concentration of Factor 4 within AG and along the RS corridors indicates that these metals are redistributed away from the mine core through deposition and land-use-specific activities rather than originating solely from direct mining emissions.

Factor 1, enriched primarily in Pb with additional contributions from Cd and Co, is indicative of traffic and residential influences.^9,66^ The association of Pb with Cd and Co, supported by their moderate correlations, suggests mixed vehicular emissions, and urban dust as contributing sources. The spatial extension of Factor 1 from coal mine areas into residential and roadside zones further supports this interpretation.

Factor 3 displayed weaker inter-element correlations and a more heterogeneous elemental profile, suggesting localized or point-source inputs rather than a single dominant process. However, its spatially restricted hotspots, particularly in the southern agricultural and coal mine areas, indicate episodic inputs that are likely associated with combustion residues, waste handling, or localized industrial activities.^67,68^ The integration of correlation structure with spatially resolved PMF outputs significantly strengthens the source attribution by demonstrating that metals grouped within each factor not only co-vary statistically but also co-occur spatially in relation to specific land-use and emission processes.

### 4.3 Predictive modelling of potentially toxic element distribution

The RF model demonstrated strong to moderate predictive capability across the investigated PTEs, confirming its suitability for modelling complex, non-linear relationships in heterogeneous mining-impacted soils. The overall average prediction accuracy reflects robust model performance, particularly given the limited sample size and strong spatial variability inherent to the study area.^69,70^ Elements such as Hg, Pb, and Cd were predicted with high accuracy and low RMSE values, indicating that their spatial distribution is strongly controlled by a combination of identifiable sources and measurable soil and land-use attributes.^21,71^

The moderate predictive performance for Cr, Ni, and Zn suggests that these elements are influenced by multiple overlapping processes, including lithogenic background contributions, mining disturbance, and post-depositional redistribution. Their higher RMSE values reflect greater spatial heterogeneity and mixed source influences rather than model inadequacy, a common limitation when predicting elements with both geogenic and anthropogenic origins in mining regions.^72^

In contrast, Co and As exhibited comparatively lower predictive accuracy, highlighting the complexity of their environmental behaviour. In the case of Co, this reduced predictability is likely related to its strong sensitivity to the soil geochemical conditions, particularly pH and redox status, which control its speciation and bioavailability.^73–75^ Similarly, As mobility is governed by redox-driven adsorption–desorption processes involving iron oxides, which are not fully captured by bulk soil physicochemical parameters alone. These findings indicate that the lower *R*^2^ values for Co and As reflect inherent geochemical complexity rather than limitations of the RF algorithm.

Feature importance analysis provided additional insight into the mechanistic drivers of PTE distribution. PMF-derived source contributions consistently ranked among the most influential predictors across multiple elements, demonstrating that incorporating quantitative source information substantially improves the model interpretability and performance. For example, the Cd and Zn predictions were strongly influenced by specific PMF factors linked to agricultural and mining activities, while Cr and Ni were primarily governed by PMF factors associated with mixed geogenic–mining sources. This confirms that source-resolved predictors capture latent processes that cannot be inferred from soil properties or spatial coordinates alone.^43^ PMF-derived source contributions are obtained from the same concentration data used as the modelling targets, introducing an inherent structural dependency when included as predictors. Therefore, PMF variables are used primarily to enhance source-specific interpretability and process understanding rather than as independent predictors, and model performance metrics are interpreted within an explanatory, source-informed framework.^76^

The inclusion of land-use variables further enhanced the prediction accuracy by accounting for anthropogenic modification of the soil environment. Residential and agricultural land-use indicators emerged as important predictors for Co, Cd, and Zn, reflecting the role of fertilizer application, traffic emissions, and secondary redistribution in shaping contamination patterns.^58,59^ Overall, the integrated PMF–RF framework advances predictive soil contamination modelling beyond purely empirical approaches by embedding source-specific information within a machine learning structure. The predictive framework in this study is primarily interpolative, as spatial patterns were derived using ordinary kriging, which explicitly accounts for spatial autocorrelation. Accordingly, the ML predictions are intended to support spatial interpretation within the sampled domain rather than extrapolation to independent regions.

### 4.4 Environmental and ecological risk assessment

The integrated pollution indices consistently indicate severe multi-metal stress in the CM and AG soils, confirming that the contamination intensity and associated ecological threats are strongly governed by land-use type and proximity to mining activities. The combined interpretation of the CF and *I*_geo_ values reveals that Co and Cd are the most persistently enriched elements across all land uses, whereas Zn shows pronounced but spatially restricted enrichment, largely confined to CM soils. This pattern demonstrates that contamination extends beyond the immediate mining footprint into the surrounding landscapes, consistent with trends reported in previous studies.^49,77,78^ In contrast, Cr consistently exhibits low CF and *I*_geo_ values despite its relatively high absolute concentrations, suggesting its dominant lithogenic origin and limited ecological mobility.^79,80^

The PLI further corroborates these findings, with values decreasing in the order of CM > AG > FT > RD > RS, confirming coal mine cores and intensively managed agricultural soils as the primary pollution hotspots. This spatial trend reflects cumulative inputs from mining-derived dust, ore handling, surface runoff, and agricultural amendments, rather than isolated point-source contamination.^81^ The convergence of multiple indices highlights that ecological risk is driven by combined metal loading rather than by a single dominant contaminant.

The ecological risk assessment reveals a clearer hierarchy of environmental threat. Cd emerges as the principal contributor to ecological risk, reaching high to very high ER levels particularly in the AG and roadside soils. This dominance is attributed to the high toxicity coefficient and strong bioavailability of Cd in biologically active soils, where it poses a direct threat to soil microorganisms and crop productivity.^82^ Hg contributes moderately to ecological risk, while Zn, Pb, Ni, Cr, and Cu generally fall within low to moderate risk categories despite their elevated concentrations. Compared with global coal-mining regions, the Cd-related risk in the CM area was higher than that reported in Jharia, India,^56^ indicating severe ecological concern. The Hg risk in the AG soils was similar to that found in Yunnan, China, suggesting comparable contamination patterns likely influenced by similar human activities.^6,83^

The spatial distribution of the PERI values reinforces the role of land use in regulating ecological vulnerability. Agricultural soils exhibit the highest cumulative ecological risk, followed by coal mine and roadside areas, whereas forest soils largely remain within low to moderate risk categories due to their higher organic matter content and reduced anthropogenic disturbance.^49,78^ These results underscore that ecological risk is not solely concentration-dependent but is strongly mediated by metal toxicity, soil properties, and land-use practices.

Overall, the environmental and ecological risk assessment demonstrates that Cd and Co are critical elements of concern in the study area, with Cd dominating ecological risk and Co contributing substantially to overall pollution pressure. The alignment among pollution indices, spatial patterns, and source apportionment confirms that mining-related activities and secondary redistribution processes jointly shape the ecological risk profiles. These findings highlight the need for land-use-specific risk mitigation strategies, particularly in agricultural zones where ecological degradation may directly translate into food-chain contamination.

### 4.5 Human health risk assessment

The human health risk assessment revealed clear age-dependent and land-use-dependent exposure patterns, with children consistently experiencing substantially higher risks than adults across all land uses. The deterministic results revealed that the HI values for children exceeded the acceptable threshold across all land uses, particularly in the CM areas, aligning with the findings of Dai *et al.*^84^ and Shen *et al.*,^85^ who also reported that children face the highest health risks from soil-bound contaminants.

Although Cr exhibited the highest individual HQ values in the deterministic calculations, the probabilistic assessment identified Co as the dominant contributor to non-carcinogenic risk, accounting for the largest fraction of children's HI. This apparent shift reflects toxicological sensitivity rather than concentration alone, as Co has a relatively low reference dose and enhanced bioavailability under the acidic soil conditions prevalent in coal mine and roadside environments.^86–88^ Consequently, even moderate Co concentrations translate into disproportionate health risks for children due to higher soil ingestion rates and lower body weight. Co exposure in children raises particular concern due to its potential cardiovascular and renal toxicity, whereas Cr remains the principal driver of CR through its established links to respiratory malignancies.^89^

CR assessment further emphasized the vulnerability of children. While the TCR values for both adults and children generally remained within or near acceptable regulatory limits, children consistently exhibited higher TCR values, approaching the upper threshold. Cr was the primary driver of carcinogenic risk, contributing approximately 81% of TCR in children, followed by smaller contributions from Cd, As, Pb, and Co. The dominance of Cr reflects its carcinogenic potency and widespread presence across all land uses, despite its comparatively low ecological mobility. These finding contrasts with many previous studies including Zhu *et al.*^90^ and Yu *et al.*,^91^ which frequently highlighted As, Pb or Cd in mining-impacted regions, and indicate that Co has likely been underestimated in conventional risk evaluations.^92,93^ Its probable origins such as mining machinery, metallurgical wear or release from coal-bearing strata underscore the need to incorporate Co more explicitly into routine monitoring protocols for mining environments.

The improved ability of probabilistic modelling to distinguish between different risk levels highlights its value in accurately identifying the key contaminants responsible for human health risks, particularly in complex mining and industrial settings where multiple pollutants interact.^94^ From a management perspective, the close correspondence between high-risk zones and CM land use indicates that prioritizing the control of mining-related emissions, vehicular activities, and waste handling practices would yield the greatest reductions in both non-carcinogenic and carcinogenic risks. The health risk assessment is based on total metal concentrations derived from strong acid digestion, which may overestimate the bioaccessible fraction of certain elements in mining-impacted soils. Accordingly, the estimated risks should be interpreted as conservative, screening-level indicators rather than absolute exposure values, particularly for elements whose bioavailability is strongly governed by mineralogical form and soil geochemistry.

### 4.6 Future research and policy recommendations

Future research should focus on bioaccessibility and speciation-based assessments of PTEs to refine exposure estimates beyond total concentrations. Integrating *in vitro* bioaccessibility tests, mineralogical characterization, and sequential extraction methods would improve the understanding of metal mobility and health relevance, particularly for Co, Cr, and Cd. Repeated temporal sampling and spatially independent validation would further strengthen the predictive capability and capture contamination dynamics under evolving mining and land-use conditions. Methodologically, spatial cross-validation and hybrid geostatistical–machine learning approaches should be explored to better account for spatial dependence and reduce optimistic bias in the model performance. Incorporating remote sensing data, dust deposition measurements, and atmospheric dispersion modelling could also enhance the early identification of emerging contamination hotspots. From a policy perspective, existing soil quality guidelines in mining regions often rely on generic thresholds that overlook land-use-specific exposure pathways and vulnerable populations, particularly children. The prominence of Co and Cd as key risk drivers highlights the mismatch between regulatory focus and actual health and ecological risks. Therefore, risk-based land-use planning, source-specific monitoring, and stricter controls on mining waste and dust emissions should be integrated into regulatory frameworks. Aligning scientific risk assessment tools with policy implementation is essential for reducing long-term environmental and public health risks in mining-impacted landscapes.

## 5. Conclusions

This study presents an integrated assessment of PTE contamination in soils surrounding an active coal mine by combining pollution indices, geostatistical analysis, source apportionment, machine learning prediction, and health risk assessment. The results demonstrate that soil contamination is strongly controlled by land use and proximity to mining activities, with coal mine and agricultural soils exhibiting the highest pollution loads and ecological stress. Pollution indices consistently identified Co and Cd as the most persistently enriched elements, while Zn showed significant but spatially confined enrichment. PMF analysis resolved four distinct sources, with mixed mining–industrial activities contributing nearly half of the total PTE contamination, and spatial mapping confirmed the propagation of mining-derived contamination into surrounding agricultural and residential areas. The integration of PMF outputs into the RF model enhanced the interpretability and prediction accuracy, yielding a strong to moderate performance (*R*^2^ ≈ 0.82) while highlighting the geochemical complexity of Co and As.

Ecological risk assessment revealed Cd as the dominant ecological threat, particularly in AG soils, whereas human health risk assessment underscored children as the most vulnerable population. Probabilistic modelling demonstrated that Co is the primary driver of NCR, while Cr dominates CR, emphasizing the importance of toxicological properties and exposure pathways in shaping risk outcomes. While the modelling framework is primarily interpolative and risk estimates are conservative due to the use of total metal concentrations, the convergence of multiple lines of evidence strengthens confidence in the identified hotspots and priority contaminants. To improve future assessments, we advocate for more extensive monitoring efforts and integrating bioavailability-adjusted health risk models to improve public health interventions and remediation strategies in similar post-industrial landscapes.

## Author contributions

Zahid Bashir: methodology, formal analysis, software, data collection, data analysis, and writing – original draft. Deep Raj: conceptualization, validation, writing – review and editing. Rangabhashiyam Selvasembian: conceptualization, validation, writing – review and editing. All authors read and approved the final manuscript.

## Conflicts of interest

The authors confirm they have no conflicts of interest that could have affected the research presented in this paper.

## Supplementary Material

RA-016-D5RA09789D-s001

## Data Availability

All data supporting this study are available from the corresponding author upon reasonable request. Supplementary information (SI) is available. See DOI: https://doi.org/10.1039/d5ra09789d.
