# Supplementary material for: Integrated machine learning and positive matrix factorization for the source-specific contamination and predictive risk assessment of potentially toxic elements in multi-land-use soils around an active coal mine
Source: RSC Adv. 2026 Feb 19;16(11):10158–78. doi: 10.1039/d5ra09789d (PMC12919393; doi:10.1039/d5ra09789d)
Supplement: RA-016-D5RA09789D-s001 [file RA-016-D5RA09789D-s001.pdf]

Supplementary Information (SI) for RSC Advances.
This journal is © The Royal Society of Chemistry 2026

Supplementary Information (SI)

Integrated machine learning and positive matrix factorization for source-specific contamination and predictive risk assessment of potentially toxic elements in multi-land-use soils around an active coal mine

Zahid Bashir[1], Deep Raj[1]✉, Rangabhashiyam Selvasembian[1,2]✉

[1]*Department of Environmental Science and Engineering, School of Engineering and Sciences, SRM University-AP, Amaravati, Andhra Pradesh 522240, India*

[2]*Centre for Interdisciplinary Research, SRM University-AP, Amaravati, Andhra Pradesh, 522240, India*

Zahid Bashir: Zahidbashir5175@gmail.com

✉Deep Raj: Email: deepraj2587@gmail.com

✉Rangabhashiyam Selvasembian: Email: rambhashiyam@gmail.com

ORCID IDs:

Zahid Bashir: 0009-0008-0490-0774

Deep Raj**:** 0000-0002-2968-1511

Rangabhashiyam Selvasembian: 0000-0003-0306-6753

Table S1. The equations and classification of I_{geo}, *PLI, EF, CF* (Fei et al., 2022; Gong et al., 2024; Hakanson et al.1980, Tomlinson et al., 1980, Huang et al., 2021, Müller et al.,1969).

Index	Equation	Category	Degree
Contamination factor (*CF*)	$CF = \frac{C_n}{S_n}$	CF < 1	Non-pollution
		1 ≤ CF < 2	Low pollution
		2 ≤ CF < 3	Moderate pollution
		3 ≤ CF < 5	High pollution
		CF ≥ 5	Extremely high pollution
Geo-accumulation index (I_{geo})	$I_{geo} = log_2\left(\frac{C_n}{1.5s_n}\right)$	I_{geo}<0	Non-pollution
		0≤I_{geo}<1	Slight pollution
		1≤I_{geo}<2	Moderate pollution
		2≤I_{geo}<3	Moderate-heavy pollution
		3≤I_{geo}<4	Heavy pollution
		4≤I_{geo}<5	Heavy-extreme pollution
		I_{geo}≥5	Extreme pollution
Pollution load index (*PLI*)	$PLI = \sqrt[n]{CF_1 \times CF_2 \times \cdots \times C}$	0 < PLI < 1	Unpolluted
		1 < PLI < 2	Moderately polluted to unpolluted
		2 < PLI < 3	Moderately polluted
		3 < PLI < 4	Moderately to highly polluted
		4 < PLI < 5	Highly polluted
		5 < PLI	Very highly polluted
Ecological risk coefficient (ER)	$ER = T_r^i \times CF$	ER < 40	Low
		40 ≤ ER< 80	Moderate
		80 ≤ ER < 160	Considerable
		160 ≤ ER < 320	High
		≥320	Very high
Potential Ecological	$PERI = \sum_{i=1}^{n} EF$	<150	Low

Risk Index (PERI)	150–300	Moderate
	300–600	Considerable
	≥600	High

Where, Cn is the concentration of PTE 'n' in the soil, and Sn is the Indian natural background value of the PTE 'n' in soil (mg/kg). T_r^i is the biological toxicity factor, T_r^i values differ for each metal: 30 for Cd, 2 for Cr, 10 for As, 40 for Hg, 1 for Zn, 5 for Cu, 5 for Pb and 5 for Ni and Co (Huang et al., 2018).

Geostatistical analysis

The spatial distribution of PTE concentrations in study area was evaluated using ordinary kriging interpolation to generate continuous prediction maps (Fig. 3). This approach provides optimal estimates at unsampled locations by integrating spatial autocorrelation and distances between sampling points, complementing the stratified random sampling design. The predicted value at an unmeasured point was calculated using Eq.:

$$Z^*(x_0) = \sum_{i=1}^{n} \lambda i Z(xi)$$

Where $Z^*(x_0)$ is the calculated value at the unobserved point x_0, $Z(xi)$ is the measured value at the known location xi, λi is the weight assigned to $Z(xi)$, and 'n' is the total number of known data points used in the interpolation process.

Source apportionment

Positive Matrix Factorization (PMF, EPA PMF v5.0) was applied to identify potential PTE sources, following USEPA guidelines. Data preprocessing included outlier removal using histograms and interquartile ranges. The PMF model decomposes the original concentration matrix as shown in Eq.:

$$X_{ij} = \sum_{k=1}^{p} g_{ik} f_{kj} + e_{ij}$$

Where, X_{ij} represents the concentration of PTE j in sample i, g_{ik} is the contribution of source k to sample i, and f_{kj} is the fraction of PTE j in source k. The model minimizes an objective function Q, which measures the overall difference between the observed data and the model's predictions, ensuring an optimal fit. The objective function Q is defined by Eq.

$$Q = \sum_{i=1}^{n}\sum_{j=1}^{m}\left(\frac{e_{ij}}{\sigma_{ij}}\right)^2$$

Where, σ_{ij} is the uncertainty associated with each data point. If the value of 'j' exceeds or is below the corresponding method detection limit (MDL), then uncertainty is estimated using Eqs.

$$Unc_{ij} = \sqrt{(S_{ij}\cdot C_{ij})^2 + (0.5\cdot MDL_j)^2}$$

$$Unc_{ij} = \frac{5}{6}\cdot MDL$$

Unc_{ij} is the uncertainty for PTE j in sample i, C_{ij} is the measured concentration (mg/kg), S_{ij} is the error fraction, and MDL_j is the method detection limit for PTE j.

Table S2. Uncertainty concentrations (mg/kg) of PTEs in land uses around Sathupalli coal mine.

PTE	Probabilistic distribution	Parameters (mean, SD)	Reference
Hg	Log normal	LN (0.9,0.6)	This study
As	Log normal	LN (8.2, 4.8)	This study
Zn	Log normal	LN (392.6, 175.8)	This study
Pb	Log normal	LN (191.9, 83.2)	This study
Co	Log normal	LN (46.9, 14.3)	This study
Cd	Log normal	LN (3.2, 1.9)	This study
Ni	Log normal	LN (148.1, 51.1)	This study
Cr	Log normal	LN (167.76, 96.0)	This study
Cu	Log normal	LN (147.9, 80.1)	This study

(MATLAB (2025b) was used to fit the probabilistic distribution of the uncertain concentrations of PTEs)

Table S3. Parameters and their detailed information for the health risk assessment (USEPA, 2011; Zhang et al., 2024; Liu et al., 2013)

Equation	Parameters	Information	Adults	Children	Units
	IR_{ing}	Ingestion rate	100	200	mg/d
	IR_{inh}	Inhalation rate	15	7.5	m^3/d
	SA	Exposed skin area	5000	1600	cm^2
	AF	Adherence factor	0.2	0.2	$mg/cm^2/d$
	ABS	Dermal absorption factor	0.001	0.001	unitless
$ADI_{ing} = \frac{C_n \times IR_{ing} \times EF \times ED \times CF}{BW \times AT}$	PEF	Particle emission factor	1.36×10^9	1.36×10^9	m^3/kg
$ADI_{der} = \frac{C_n \times SA \times AF \times ABS \times EF \times ED \times}{BW \times AT}$	EF	Exposure frequency	365	350	d/a
$ADI_{inh} = \frac{C_n \times IR_{inh} \times EF \times ED}{PEF \times BW \times AT}$	ED	Exposure duration	30	6	a
	BW	Average body weight	55.9	15.9	kg
	AT	Average time: Non-carcinogenic	365×ED	365×ED	d
		Average time: Carcinogenic	365×70	365×70	
	CF	Unit conversion factor	0.000001	0.000001	kg/mg

Table S4. Parameters for the exposure risk calculations of PTEs in soil with Mote Carlo simulation.

Description	Parameter	Unit	Type	Children	Adult	Reference
Exposure frequency	EF	day/year	Triangular	TRI (180, 345, 365)		(Huang et al., 2021)
Ingestion rate of soil	IR_{ing}	mg/day	Triangular	TRI (66, 103, 161)	TRI (4, 30, 52)	(Yang et al., 2019)
Skin adherence factor	AF	mg/cm^2	Lognormal	LN (0.65, 1.2)	LN (0.49, 0.54)	(Chen et al., 2019)

Inhalation rate	IR_{inh}	m^3/day	Point	7.5	14.5	(Han et al., 2020)
Exposure duration	ED	years	Point	6	24	(Han et al., 2020)
Particle emission factor	PEF	m^3/kg	Point	1.36×10^9	1.36×10^9	(Huang et al., 2021)
Average body weight	BW	kg	Point	15	55.9	(Han et al., 2020)
Average time of exposure to contaminated soils	AT	day	Point	$365 \times ED$（non-carcinogenic）<br>365×70（carcinogenic）		(Han et al., 2020)
Exposed skin area	SA	m^2	Triangular	TRI(0.076, 0.086, 0.382)	TRI(0.076, 0.153, 0.382)	(Huang et al., 2018)
Dermal adsorption factor	ABS	-	-	0.001（non-carcinogenic）<br>0.01（carcinogenic）		(Huang et al., 2021)

Table S5. The reference doses (*RfD*) and slope factors (*SF*). (*RfD* for noncarcinogenic PTEs and SF for carcinogenic PTEs).

PTE	*RfD* (mg/kg·d)			*SF* (kg/d·mg)		
	RfD_{ing}	RfD_{inh}	RfD_{derm}	SF_{ing}	SF_{inh}	SF_{derm}
As	3.00E-04[a]	1.23E-04[a]	1.23E-04[a]	1.50E+00[a]	1.51E+01[a]	3.66E+00[a]
Cd	1.00E-03[a]	1.00E-05[a]	1.00E-05[a]	6.10E+00[a]	6.30E+00[a]	3.80E-01[b]
Co	2.00E-02[c]	1.60E-03[c]	5.71E-06[c]	N/A	N/A	9.80E+00[c]
Cu	4.00E-02[a]	4.02E-02[a]	1.20E-02[a]	N/A	N/A	N/A
Ni	2.00E-02[a]	2.06E-02[a]	5.40E-03[a]	1.70E+00[e]	9.00E-01[e]	4.25E+00[e]
Pb	3.50E-03[a]	3.52E-03[a]	5.25E-04[a]	8.50E-03[a]	N/A	N/A
Zn	3.00E-01[a]	3.00E-01[a]	6.00E-02[a]	N/A	N/A	N/A
Hg	3.00E-04	8.57E-05[d]	2.10E-05[d]	N/A	N/A	NA
Cr	3.00E-03[e]	2.86E-05[e]	6.00E-05[e]	8.50E-03[f]	4.20E+01[f]	2.00E+01[g]

N/A, Data not available, [a](Huang et al., 2021), [b](Wang et al., 2017), [c](Han et al., 2020), [d](Lei et al., 2022) [e](Tong et al., 2020;), [f](Zhou et al.,2022), [g](Wang et al., 2020).

Table S6. The classification of *HI* and *TCR* (*CR*) (US EPA, 2001)

Category	Degree
HI <1	No risk of adverse health effects
HI ≥1	The occurrence of non-carcinogenic effects.
CR ≤ 1.00E-06	A mild risk
1.00E-06 ≤ (*CR*) ≤ 1.00E-04	Acceptable risk
TCR (*CR*) ≥ 1.00E-04	Unacceptable risk

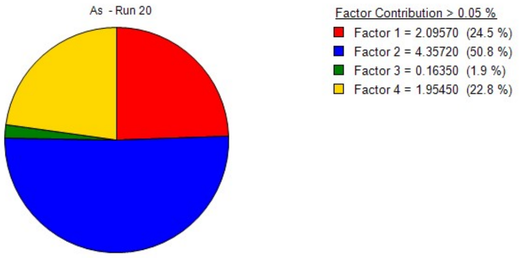
As - Run 20
Factor Contribution > 0.05 %
Factor 1 = 2.09570 (24.5 %)
Factor 2 = 4.35720 (50.8 %)
Factor 3 = 0.16350 (1.9 %)
Factor 4 = 1.95450 (22.8 %)

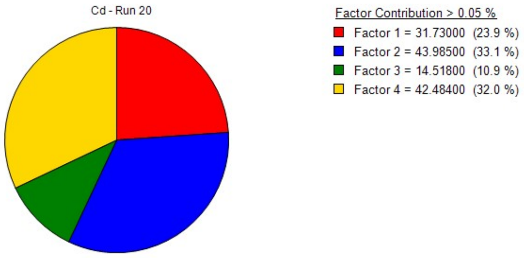
Cd - Run 20
Factor Contribution > 0.05 %
Factor 1 = 31.73000 (23.9 %)
Factor 2 = 43.98500 (33.1 %)
Factor 3 = 14.51800 (10.9 %)
Factor 4 = 42.48400 (32.0 %)

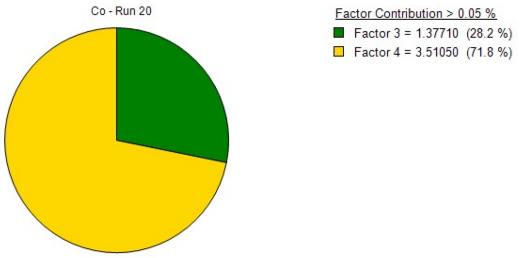
Co - Run 20
Factor Contribution > 0.05 %
Factor 3 = 1.37710 (28.2 %)
Factor 4 = 3.51050 (71.8 %)

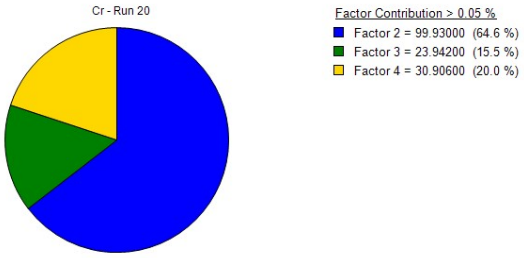
Cr - Run 20
Factor Contribution > 0.05 %
Factor 2 = 99.93000 (64.6 %)
Factor 3 = 23.94200 (15.5 %)
Factor 4 = 30.90600 (20.0 %)

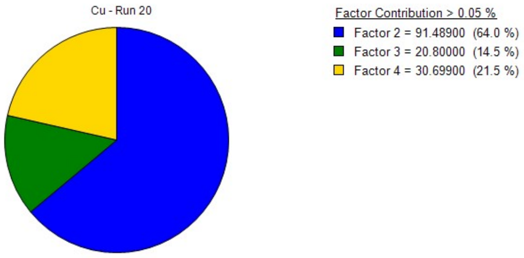
Cu - Run 20
Factor Contribution > 0.05 %
Factor 2 = 91.48900 (64.0 %)
Factor 3 = 20.80000 (14.5 %)
Factor 4 = 30.69900 (21.5 %)

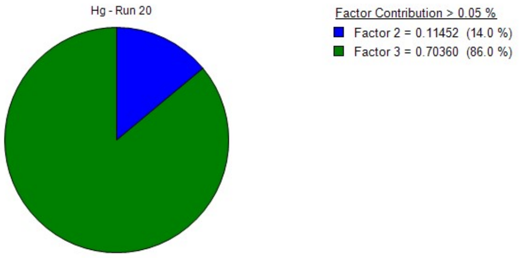
Hg - Run 20
Factor Contribution > 0.05 %
Factor 2 = 0.11452 (14.0 %)
Factor 3 = 0.70360 (86.0 %)

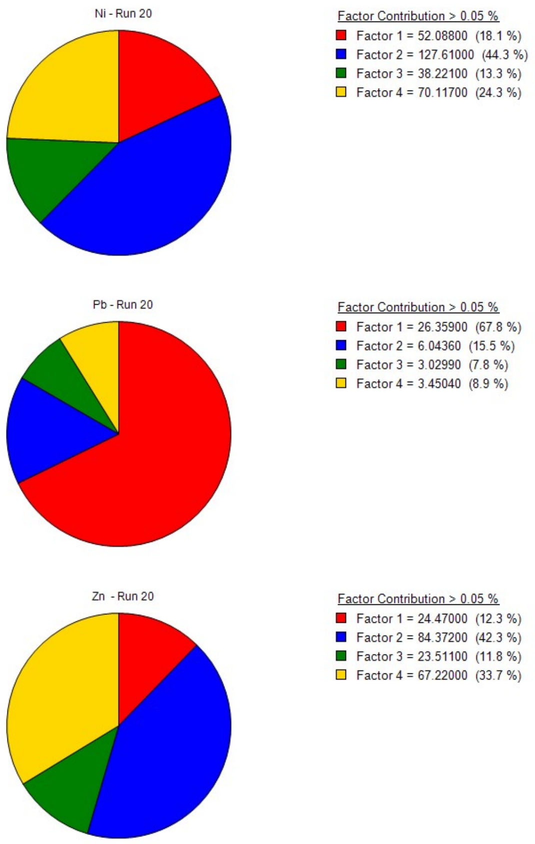

Fig. S1 Contribution of PTEs to each factor.

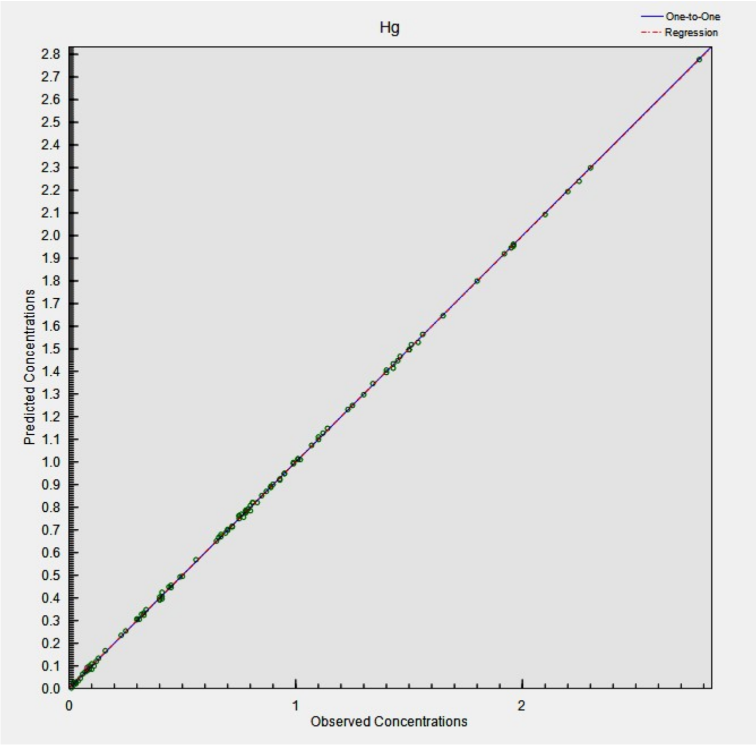
Hg
One-to-One
Regression
Predicted Concentrations
Observed Concentrations

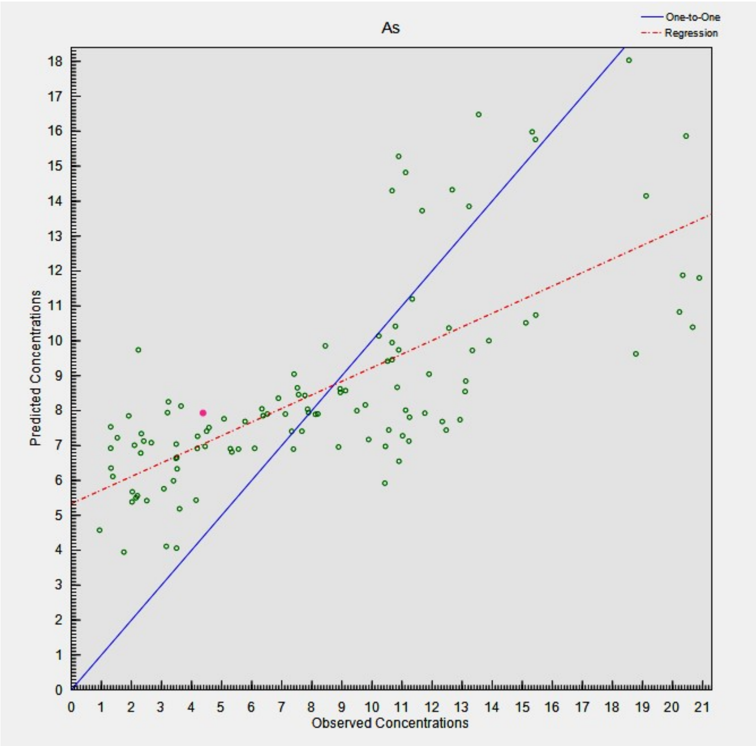
As
One-to-One
Regression
Predicted Concentrations
Observed Concentrations

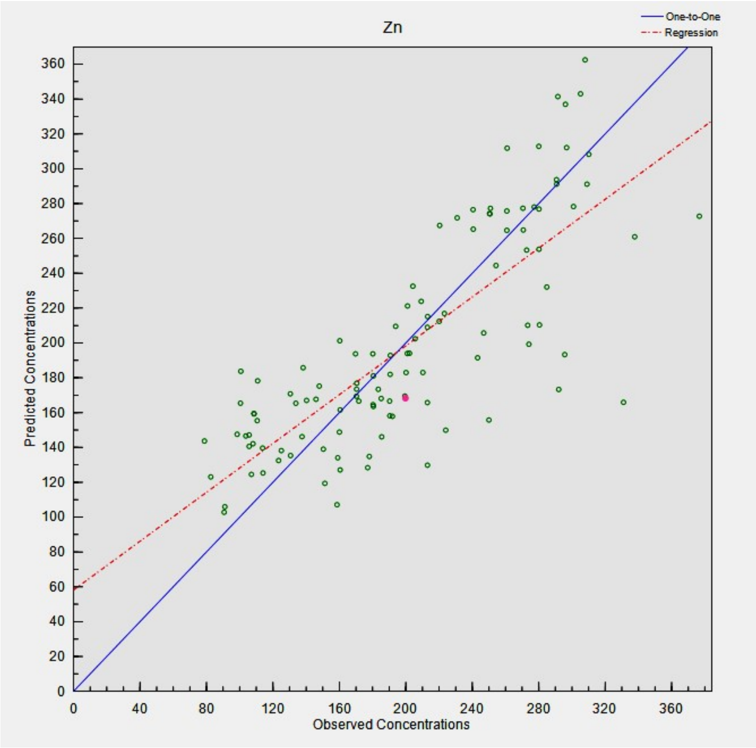
Zn
One-to-One
Regression
Predicted Concentrations
Observed Concentrations
0
20
40
60
80
100
120
140
160
180
200
220
240
260
280
300
320
340
360
0
40
80
120
160
200
240
280
320
360

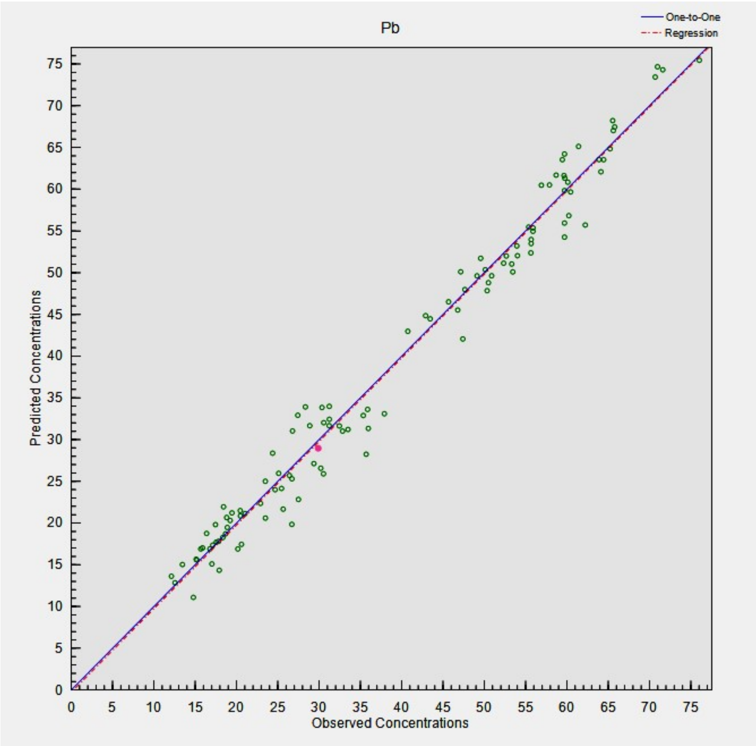
Pb
One-to-One
Regression
Predicted Concentrations
Observed Concentrations
0
5
10
15
20
25
30
35
40
45
50
55
60
65
70
75

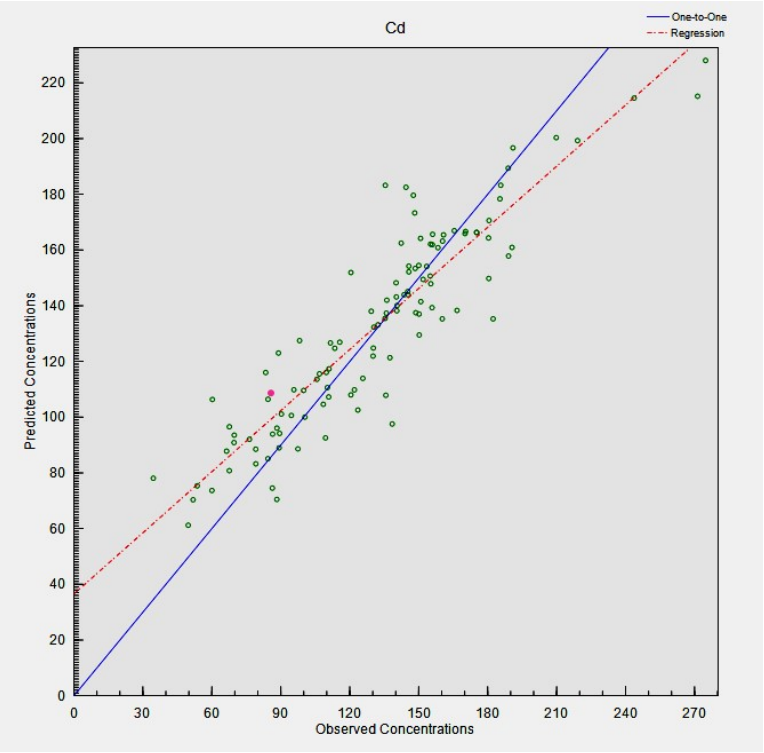
Cd
One-to-One
Regression
Predicted Concentrations
Observed Concentrations
0
20
40
60
80
100
120
140
160
180
200
220
0
30
60
90
120
150
180
210
240
270

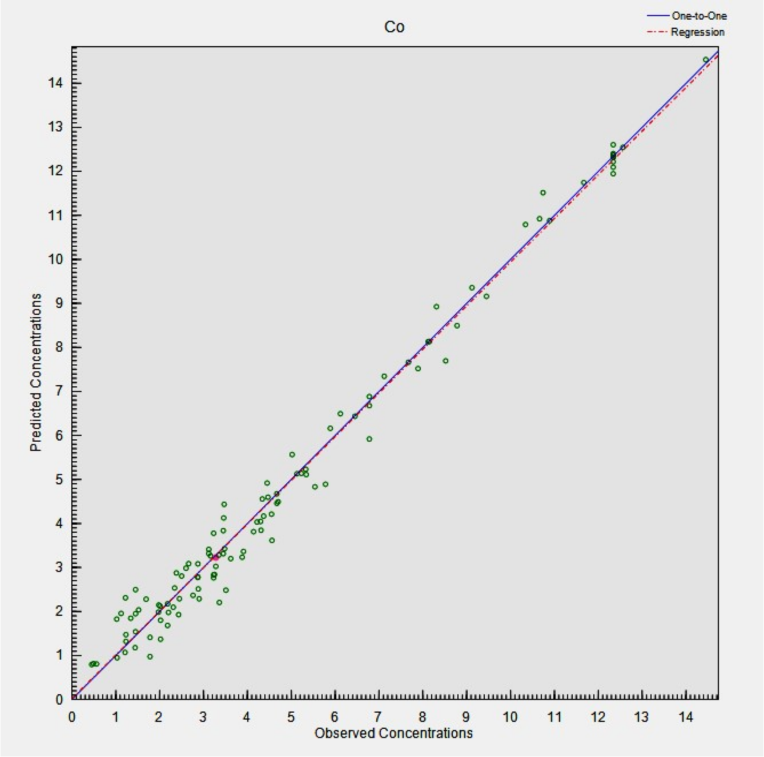
Co
One-to-One
Regression
Predicted Concentrations
Observed Concentrations
0
1
2
3
4
5
6
7
8
9
10
11
12
13
14
0
1
2
3
4
5
6
7
8
9
10
11
12
13
14

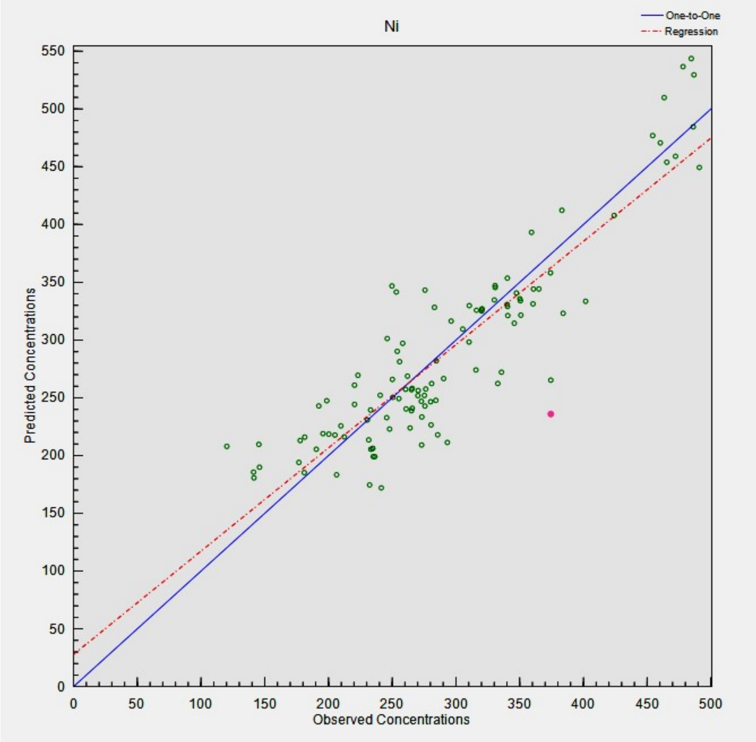

Ni
One-to-One
Regression
Predicted Concentrations
Observed Concentrations
0
50
100
150
200
250
300
350
400
450
500
550

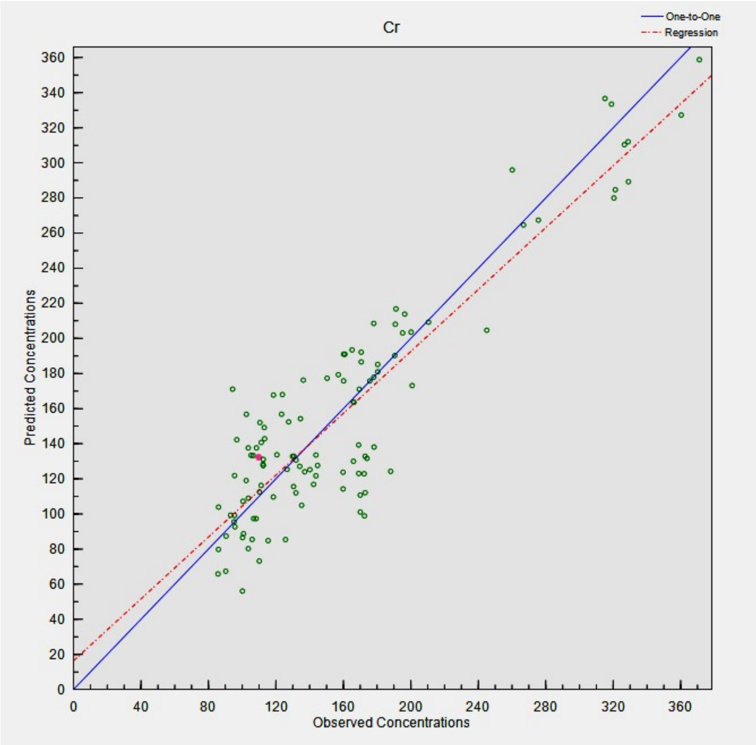

Cr
One-to-One
Regression
Predicted Concentrations
Observed Concentrations
0
20
40
60
80
120
160
200
240
280
320
360

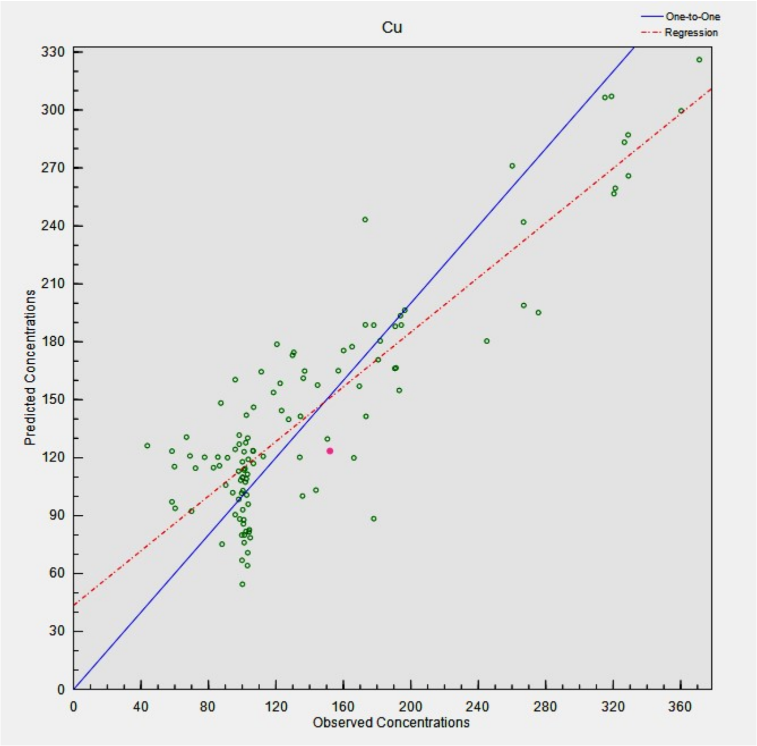

Fig. S2 Fitting coefficients (r^2) between soil PTEs observed concentrations and predicted concentrations by PMF model.

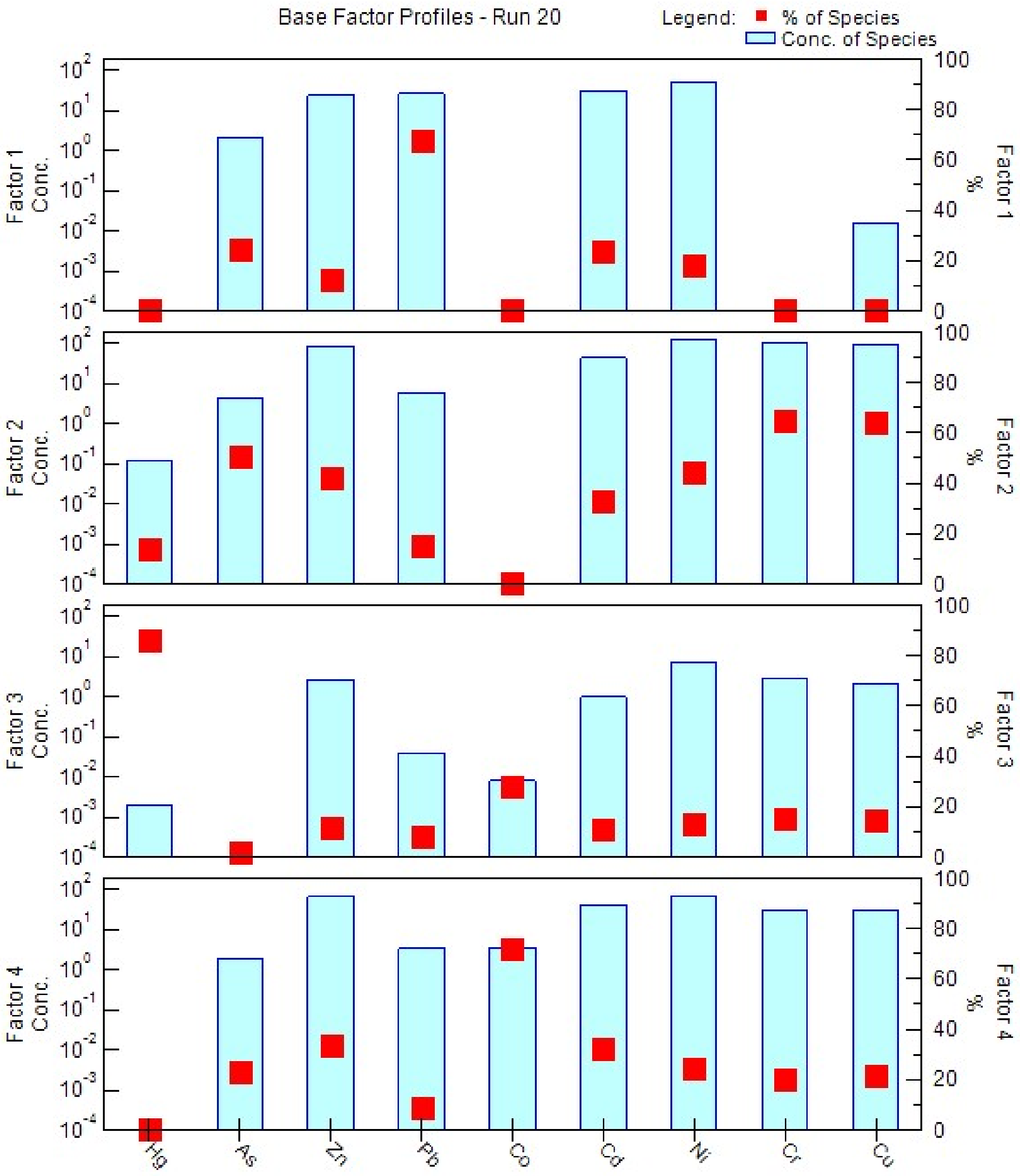

Fig. S3 Factor profile of four factors (i.e., pollution sources) from the PMF model to eight PTEs is shown. The minimum and maximum displacements are shown in the boxes, and the factor contributions obtained in the base displacements are shown as filled blue boxes for comparison, red square was % of species (i.e., PTE).

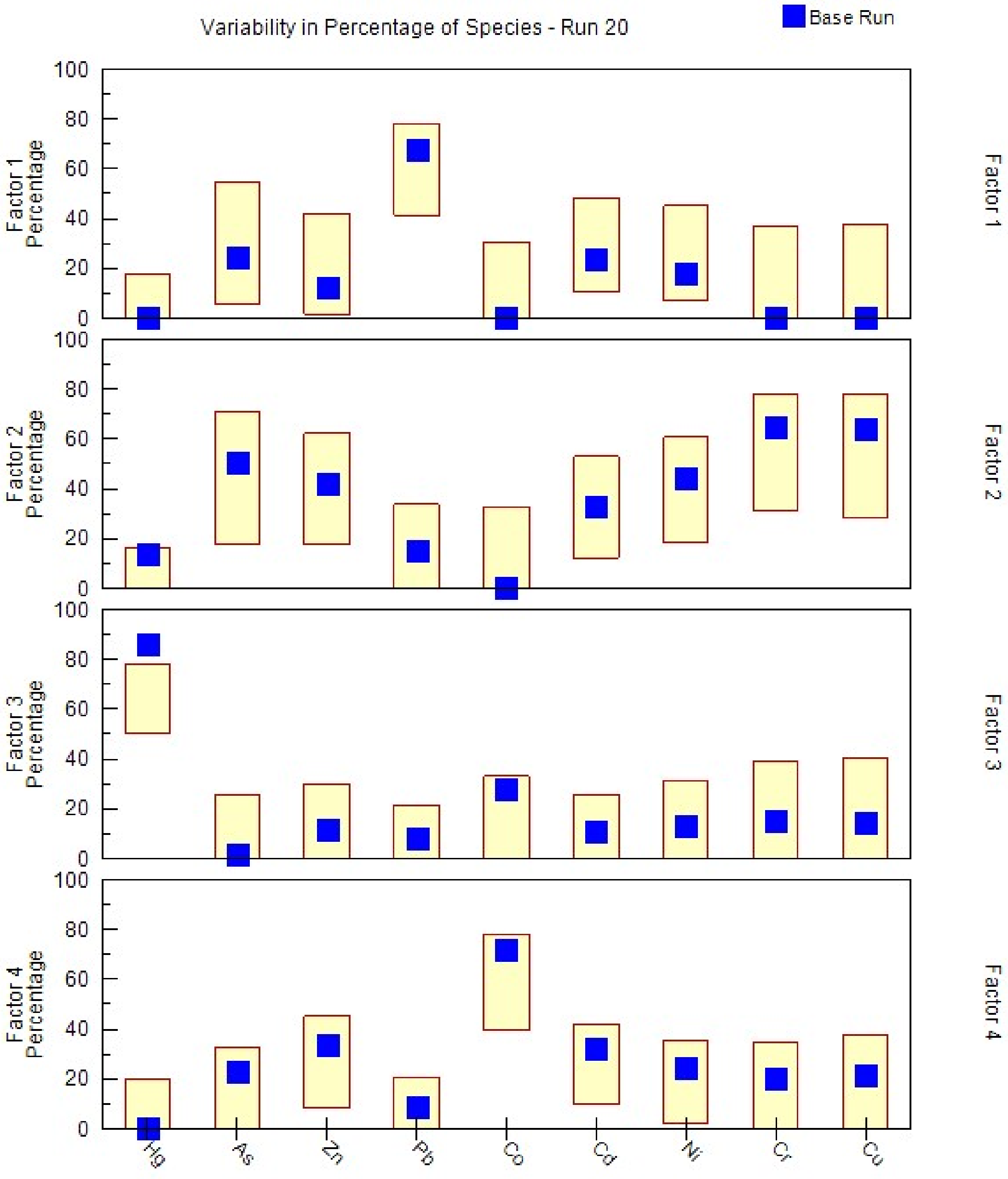

Fig. S4. Based on DISP analysis, the variability of the percentage contribution of four factors (i.e., pollution sources) from the PMF model to eight PTEs is shown. The minimum and maximum displacements are shown in the boxes, and the factor contributions obtained in the base displacements are shown as filled blue boxes for comparison.

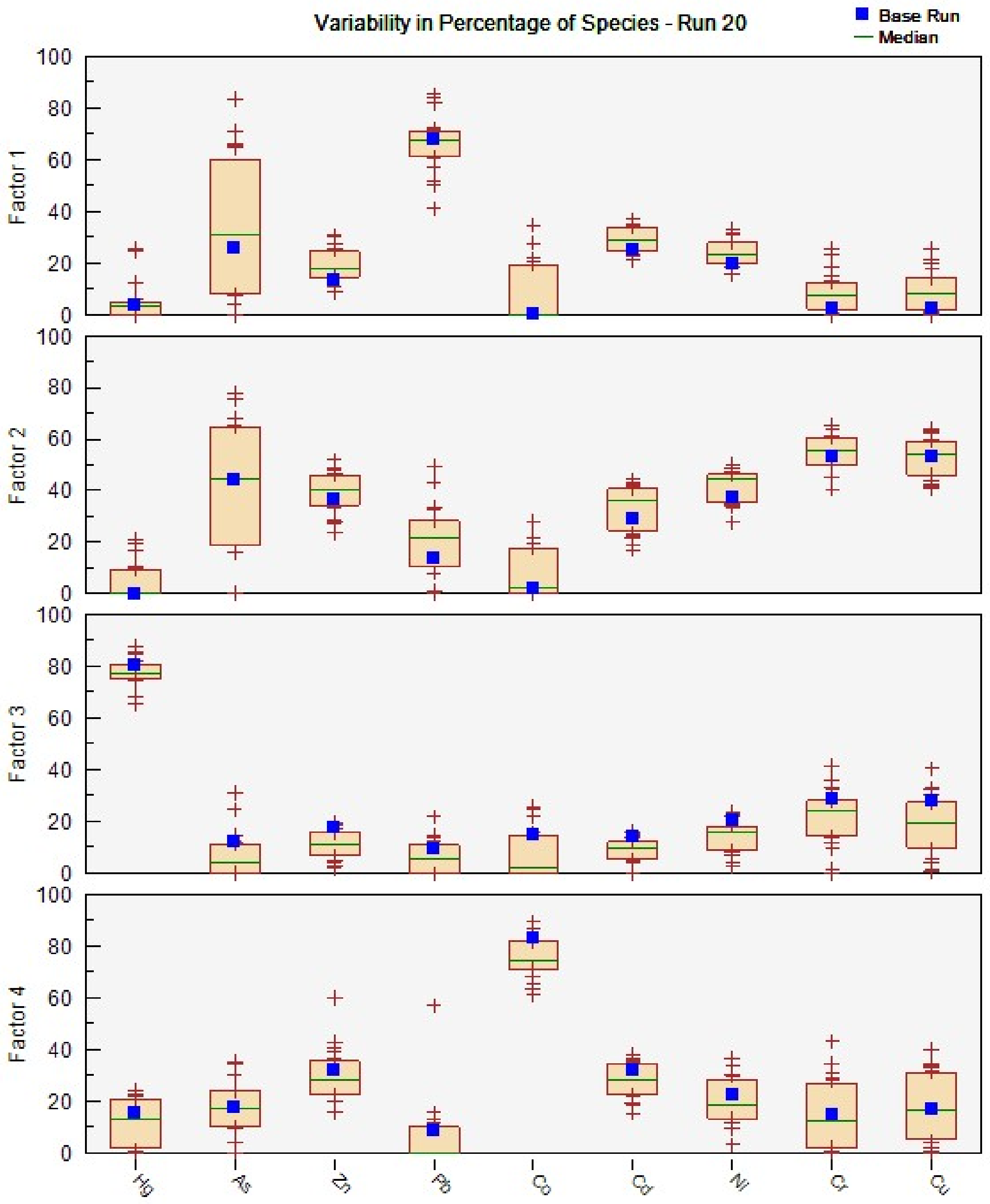

Fig. S5a. Based on Bootstrap (BS) analysis, the variability of the percentage contribution of four factors (i.e., pollution sources) from the PMF model to eight PTEs is shown. The box represents the interquartile range (25th to 75th percentile), and values outside this range are represented as crosses. The horizontal green line represents the median, and the percentage factor contribution obtained in the base test is shown as a filled blue box for comparison.

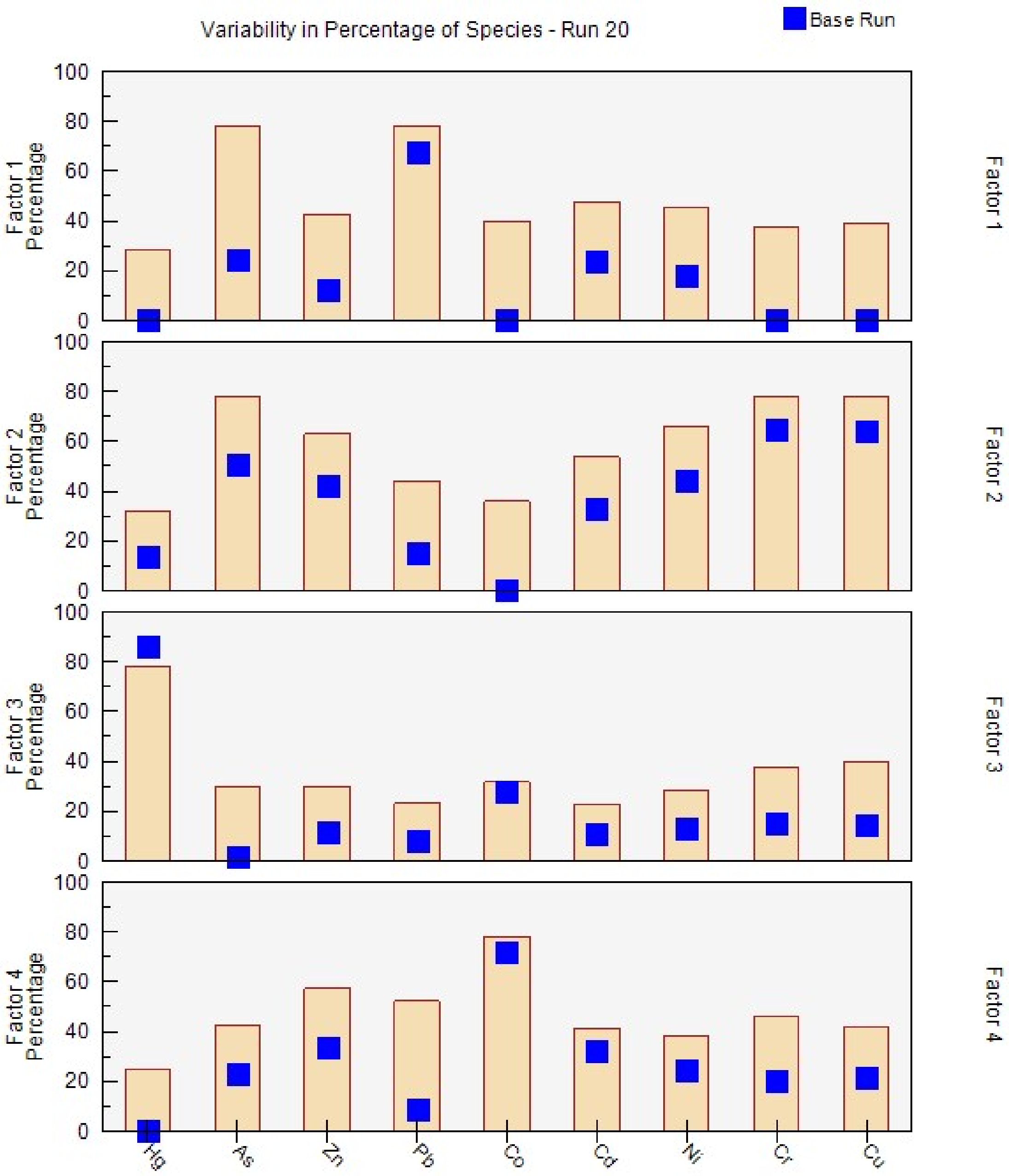

Fig. S5b. Based on Bootstrap-DISP analysis variability in species contributions to PMF Factors 1–4. The bars represent the range of percent contribution for each metal, while blue squares indicate the base-run values. This plot reflects model stability and the robustness of species–factor associations.

Table S7. Non-Carcinogenic and carcinogenic health risk assessment of potentially toxic element in different landuses around SCM.

HQ by PTEs – Adults										
**Land Use**	**Hg**	**As**	**Zn**	**Pb**	**Co**	**Cd**	**Ni**	**Cr**	**Cu**	**HI**
**CM**	7.70E-03	4.72E-02	3.10E-03	1.18E-01	1.46E-01	1.17E-02	1.71E-02	3.38E-01	9.30E-03	**6.83E-01**
**RD**	5.60E-03	2.42E-02	1.60E-03	9.05E-02	1.84E-01	1.33E-02	1.37E-02	2.61E-01	6.40E-03	**5.97E-01**
**AG**	6.00E-03	3.53E-02	2.00E-03	9.24E-02	1.62E-01	1.12E-02	1.13E-02	2.07E-01	4.20E-03	**5.32E-01**
**FT**	4.40E-03	8.89E-02	2.60E-03	7.67E-02	1.50E-01	8.20E-03	8.50E-03	1.94E-01	3.90E-03	**5.38E-01**
**RS**	1.30E-03	7.20E-02	1.90E-03	1.19E-01	1.31E-01	1.26E-02	1.10E-02	2.25E-01	5.50E-03	**5.73E-01**
**HQ by PTEs – Children**										
**CM**	4.64E-02	3.12E-01	2.01E-02	7.53E-01	1.80E-01	4.55E-02	1.12E-01	1.63E+00	6.07E-02	**3.10E+00**
**RD**	3.40E-02	1.60E-01	1.06E-02	5.78E-01	2.27E-01	5.20E-02	8.97E-02	1.26E+00	4.18E-02	**2.44E+00**
**AG**	3.60E-02	2.33E-01	1.31E-02	5.91E-01	2.01E-01	4.36E-02	7.36E-02	9.99E-01	2.78E-02	**2.22E+00**
**FT**	2.67E-02	5.87E-01	1.71E-02	4.90E-01	1.86E-01	3.21E-02	5.58E-02	9.38E-01	2.53E-02	**2.36E+00**
**RS**	7.60E-03	4.76E-01	1.24E-02	7.60E-01	1.62E-01	4.91E-02	7.22E-02	1.08E+00	3.62E-02	**2.65E+00**

Cancer Risk (CR) by PTEs – Adults							
**Land Use**	**As**	**Pb**	**Co**	**Cd**	**Ni**	**Cr**	**Total CR**
**CM**	9.10E-06	1.41E-06	3.39E-06	1.52E-05	2.46E-04	6.13E-05	**2.14E-03**
**RD**	4.67E-06	1.08E-06	4.28E-06	1.74E-05	1.98E-04	4.73E-05	**1.74E-03**
**AG**	6.82E-06	1.10E-06	3.79E-06	1.45E-05	1.62E-04	3.75E-05	**1.43E-03**
**FT**	1.72E-05	9.17E-07	3.50E-06	1.07E-05	1.23E-04	3.52E-05	**1.29E-03**
**RS**	1.39E-05	1.42E-06	3.05E-06	1.64E-05	1.59E-04	4.07E-05	**1.50E-03**
**Cancer Risk (CR) by PTEs – Children**							
**Land Use**	**As**	**Pb**	**Co**	**Cd**	**Ni**	**Cr**	**Total CR**
**CM**	1.20E-05	1.90E-06	7.32E-07	2.05E-05	3.25E-04	1.62E-05	**5.74E-03**
**RD**	6.17E-06	1.46E-06	9.24E-07	2.34E-05	2.61E-04	1.25E-05	**4.75E-03**
**AG**	9.00E-06	1.49E-06	8.17E-07	1.96E-05	2.14E-04	9.88E-06	**3.95E-03**
**FT**	2.27E-05	1.24E-06	7.56E-07	1.44E-05	1.62E-04	9.28E-06	**3.41E-03**
**RS**	1.83E-05	1.92E-06	6.57E-07	2.21E-05	2.10E-04	1.07E-05	**4.10E-03**

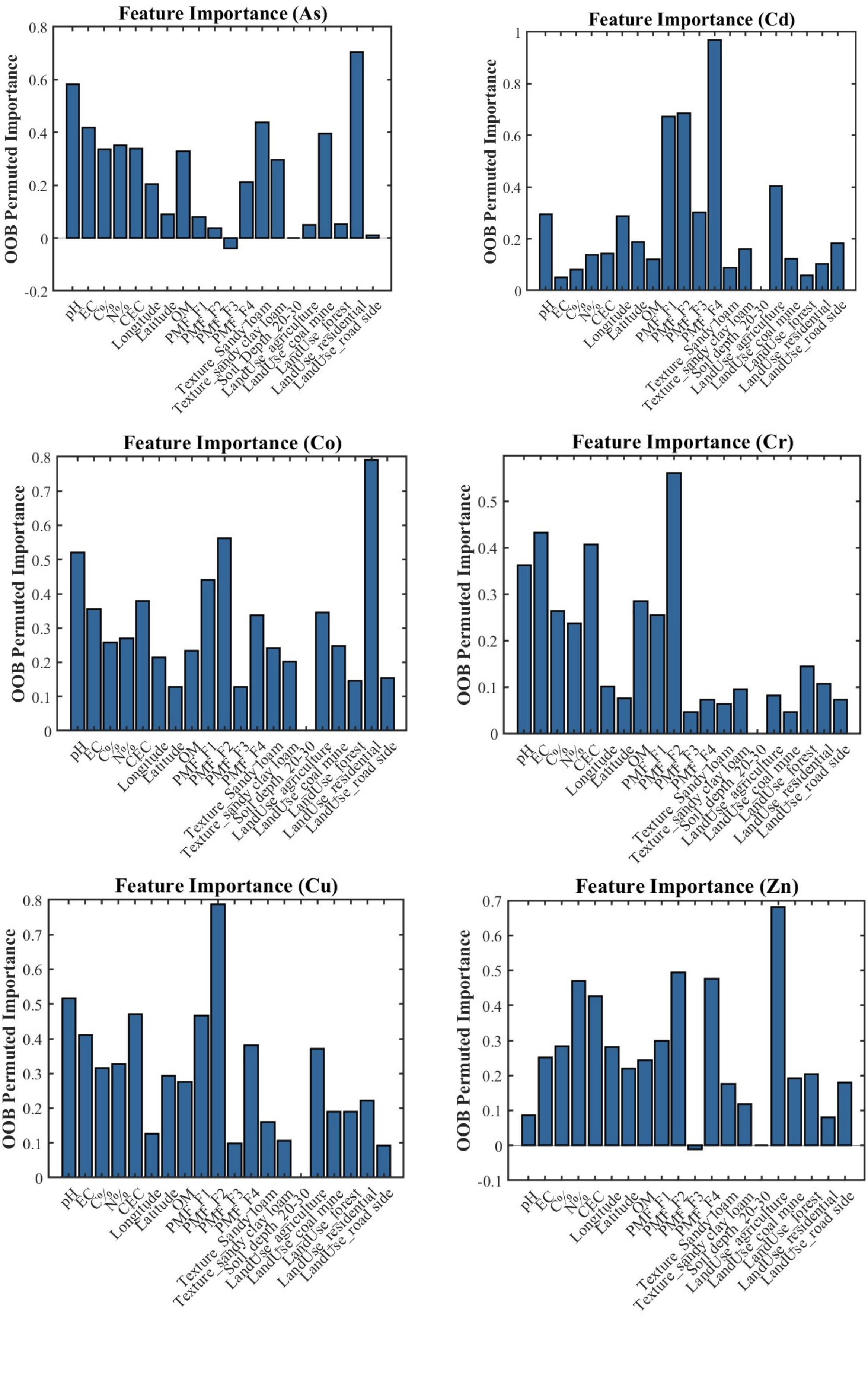

Feature Importance (As)
Feature Importance (Cd)
Feature Importance (Co)
Feature Importance (Cr)
Feature Importance (Cu)
Feature Importance (Zn)
OOB Permuted Importance
pH
EC
C%
N%
CEC
Longitude
Latitude
OM
PMF_F1
PMF_F2
PMF_F3
PMF_F4
Texture_Sandy loam
Texture_sandy clay loam
Soil depth_20-30
LandUse_agriculture
LandUse_coal mine
LandUse_forest
LandUse_residential
LandUse_road side

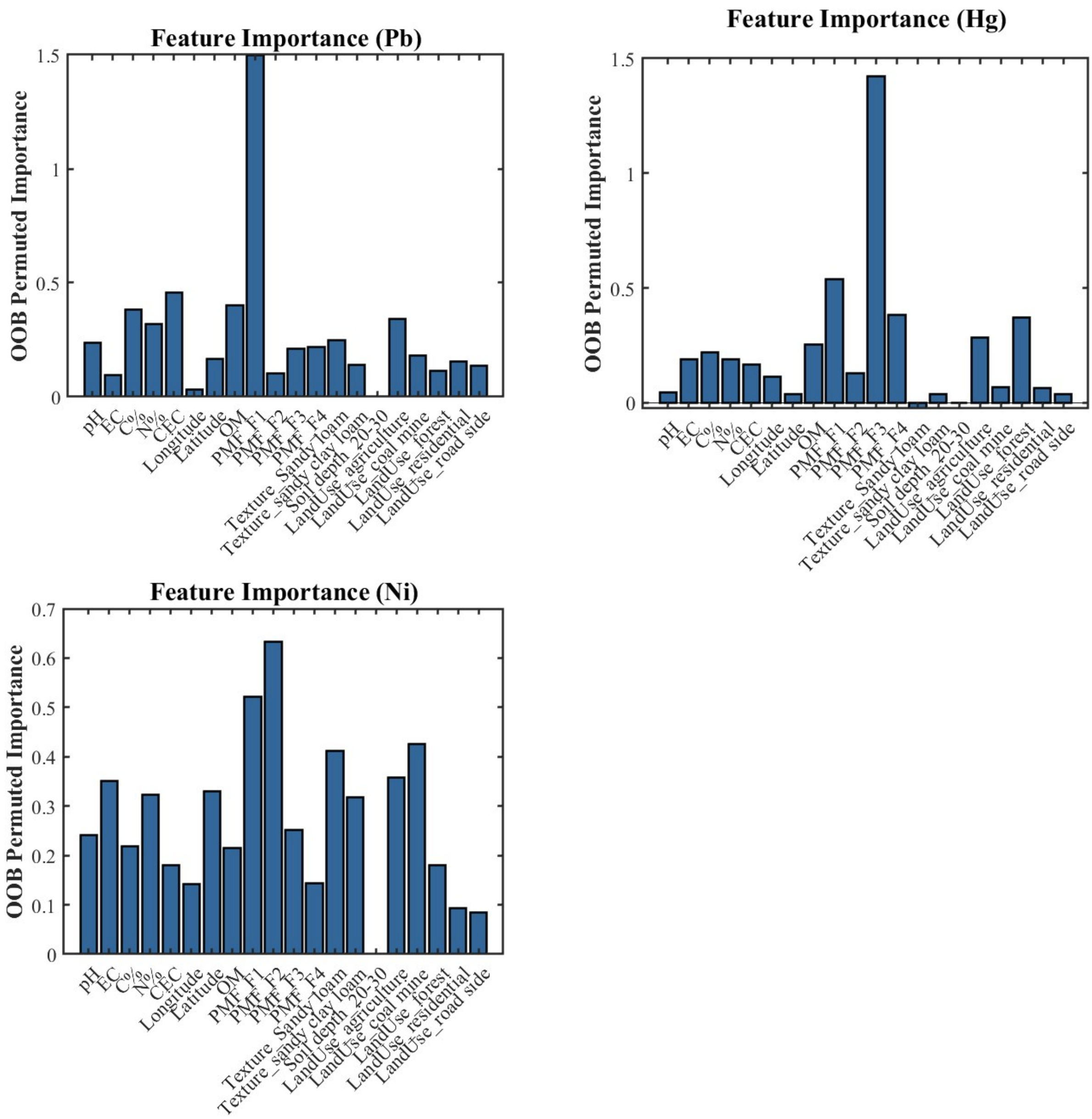

Fig. S6: Feature importance plot showing the relative contribution of physicochemical properties, PMF factors, soil texture, and land-use categories in predicting PTE concentrations. Higher OOB-permuted importance values indicate stronger predictive influence of each variable.

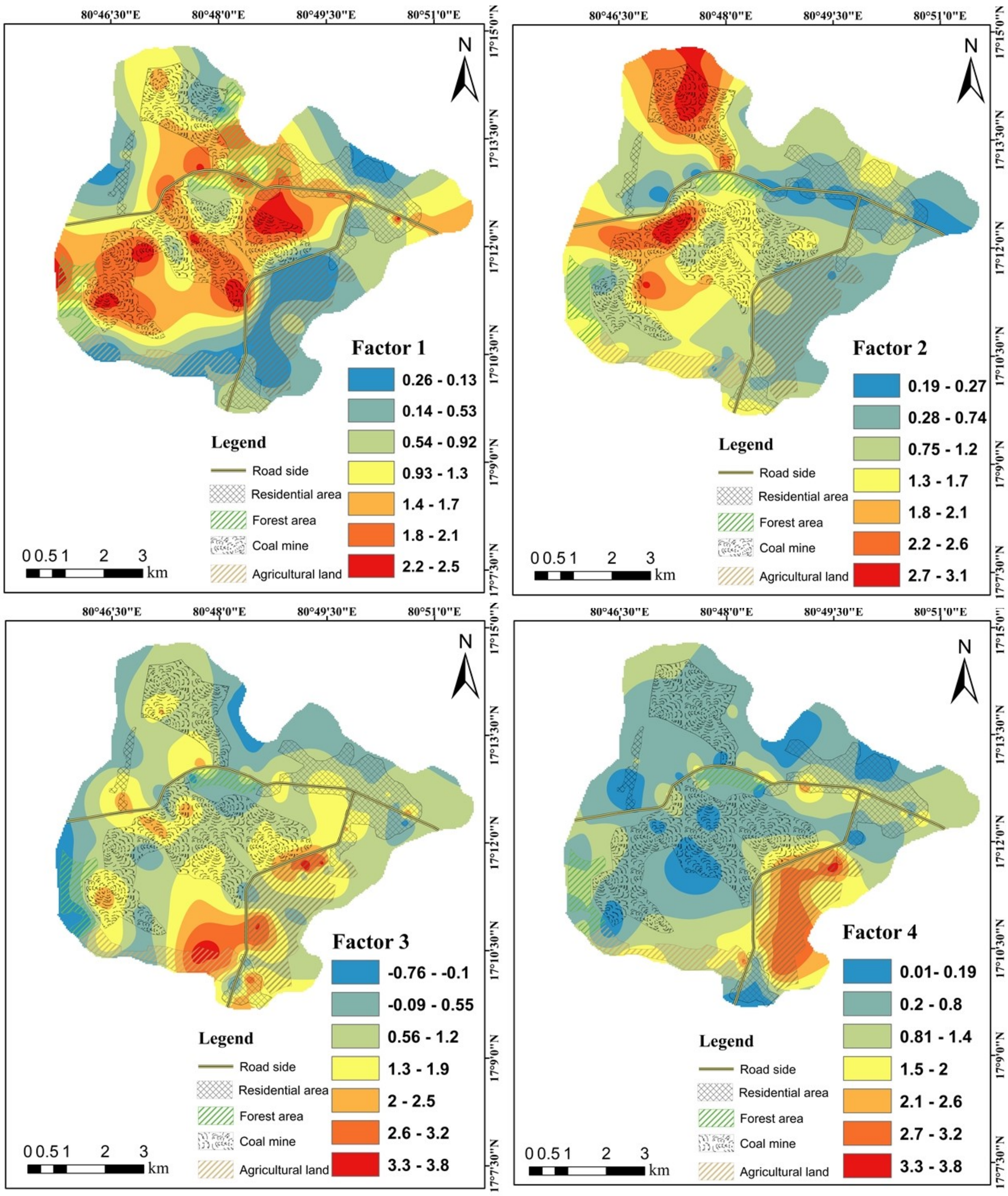

Fig. S7. Factor contributions of PTEs derived from the PMF model.

Table S8. Performance metrics of the RF model for predicting potentially toxic element concentrations, including R^2, Q^2, and RMSE (mg kg^{-1}) for each element.

Element	R^2	Q^2	RMSE (mg kg^{-1})
Hg	0.95	0.95	0.14
As	0.74	0.73	2.78
Zn	0.75	0.59	35.7
Pb	0.91	0.92	5.03
Co	0.74	0.74	23.92
Cd	0.87	0.82	1.16
Ni	0.78	0.70	44.02
Cr	0.80	0.81	38.38
Cu	0.86	0.87	25.6

Table S9. Semi-variogram parameters of the fitted models for selected PTEs.

Element	Model type	Nugget (C_0)	Sill (C_0 + C)	Range (m)	Nugget/Sill (%)
Hg	Spherical	0.22	0.52	870	42
As	Spherical	0.06	0.20	670	30
Zn	Spherical	0.11	0.42	700	26
Pb	Spherical	0.27	0.82	1080	33
Co	Spherical	0.46	1.30	980	35
Cd	Spherical	0.09	0.30	800	30
Ni	Spherical	0.14	0.38	850	37
Cr	Spherical	0.03	0.08	520	38
Cu	Spherical	0.11	0.42	820	26

References

Chen, R.H., Chen, H.Y., Song, L.T., Yao, Z.P., Meng, F.S., Teng, Y.G., 2019. Characterization and source apportionment of heavy metals in the sediments of Lake Tai (China) and its surrounding soils. Sci Total Environ 694.

Fei, X., Lou, Z., Xiao, R., Ren, Z. and Lv, X., 2022. Source analysis and source-oriented risk assessment of heavy metal pollution in agricultural soils of different cultivated land qualities.Journal of Cleaner Production,341, p.130942.

Gong, C., Quan, L., Chen, W., Tian, G., Zhang, W., Xiao, F. and Zhang, Z., 2024. Ecological risk and spatial distribution, sources of heavy metals in typical purple soils, southwest China.Scientific Reports,14(1), p.11342.

Han, Q., Wang, M., Cao, J., Gui, C., Liu, Y., He, X., He, Y., Liu, Y., 2020. Health risk assessment and bioaccessibilities of heavy metals for children in soil and dust from urban parks and schools of Jiaozuo, China. Ecotoxicol Environ Saf 191, 110157.

Huang, J.H., Guo, S.T., Zeng, G.M., Li, F., Gu, Y.L., Shi, Y.H., Shi, L.X., Liu, W.C., Peng, S.Y., 2018. A new exploration of health risk assessment quantification from sources of soil heavy metals under different land use. Environ Pollut 243, 49-58.

Hakanson, L. (1980). An ecological risk index for aquatic pollution control.a sedimentological approach. Water Research, 14(8), 975–1001.

Huang, J.L., Wu, Y.Y., Sun, J.X., Li, X., Geng, X.L., Zhao, M.L., Sun, T., Fan, Z.Q., 2021. Health risk assessment of heavy metal(loid)s in park soils of the largest megacity in China by using Monte Carlo simulation coupled with Positive matrix factorization model. Journal of Hazardous Materials 415, 11.

Huang, J.H., Guo, S.T., Zeng, G.M., Li, F., Gu, Y.L., Shi, Y.H., et al., 2018. A new exploration of health risk assessment quantification from sources of soil heavy metals under different land use. Environ. Pollut. 243, 49–58.

Müller, G., 1969. Index of geoaccumulation in sediments of the Rhine Rive. Geojournal 2, 108-118.

Lei, M., Li, K., Guo, G.H., Ju, T.N., 2022. Source-specific health risks apportionment of soil potential toxicity elements combining multiple receptor models with Monte Carlo simulation. Science of the Total Environment 817, 15.

Tong, S., Li, H., Wang, L., Tudi, M., Yang, L., 2020. Concentration, spatial distribution, contamination degree and human health risk assessment of heavy metals in urban soils across China between 2003 and 2019-A systematic review. Int J Environ Res Public Health 17.

Tomlinson, D. L., Wilson, J. G., Harris, C. R., & Jeffrey, D. W. (1980). Problems in the assessment of heavy-metal levels in estuaries and the formation of a pollution index. Helgoländer Meeresuntersuchungen, 33(1–4), 566–575.

Wang, G., Liu, H.Q., Gong, Y., Wei, Y., Miao, A.J., Yang, L.Y., Zhong, H., 2017. Risk assessment of metals in urban soils from a typical industrial city, Suzhou, Eastern China. Int J Environ Res Public Health 14.

Wang, X. F., Deng, C. B., Sunahara, G., Yin, J., Xu, G. P., Zhu, K. X., 2020. Risk assessments of heavy metals to children following non-dietary exposures and sugarcane consumption in a rural area in Southern China. Exposure and Health, 12(1), 1-8. doi.org/10.1007/s12403-018-0275-0

Yang, S.Y., Zhao, J., Chang, S.X., Collins, C., Xu, J.M., Liu, X.M., 2019. Status assessment and probabilistic health risk modeling of metals accumulation in agriculture soils across China: A synthesis. Environ Int 128, 165-174.

Zhou, Y., Jiang, D.D., Ding, D., Wu, Y.J., Wei, J., Kong, L.Y., Long, T., Fan, T.T., Deng, S.P., 2022. Ecological-health risks assessment and source apportionment of heavy metals in agricultural soils around a super-sized lead-zinc smelter with a long production history, in China. Environmental Pollution 307, 11.
